# Likelihood of atom–atom contacts in crystal structures of halogenated organic compounds

**DOI:** 10.1107/S2052252515003255

**Published:** 2015-04-10

**Authors:** Christian Jelsch, Sarra Soudani, Cherif Ben Nasr

**Affiliations:** a CRM2, CNRS, Institut Jean Barriol, Université de Lorraine, Vandoeuvre les Nancy CEDEX, France; b Faculté des Sciences de Bizerte, Laboratoire de Chimie des Matériaux, Université de Carthage, Zarzouna 7021, Tunisia

**Keywords:** intermolecular contacts, halogenated organic compounds, halogen bonding, π-stacking interactions, crystal packing, Hirshfeld surface analysis, enrichment ratio

## Abstract

The likelihood of occurrence of intermolecular contacts in crystals of halogenated organic compounds has been analysed statistically using tools based on the Hirshfeld surface. Several families of small halogenated molecules (containing organic F, Cl, Br or I atoms) were analysed, based on chemical composition and aromatic or aliphatic character. The behaviour of crystal contacts was also probed for molecules containing O or N. So-called halogen bonding (a halogen making short interactions with O or N, or a π interaction with C) is generally disfavoured, except when H is scarce on the molecular surface. Similarly, halogen⋯halogen contacts are more rare than expected, except for molecules that are poor in H. In general, the H atom is found to be the preferred partner of organic halogen atoms in crystal structures. On the other hand, C⋯C interactions in parallel π-stacking have a high propensity to occur in halogenated aromatic molecules. The behaviour of the four different halogen species (F, Cl, Br, I) is compared in several chemical composition contexts. The analysis tool can be refined by distinguishing several types for a given chemical species, such as H atoms bound to O or C. Such distinction shows, for instance, that C—H⋯Cl and O—H⋯O are the preferred interactions in compounds containing both O and Cl.

## Abstract 2

The analysis, based on Hirshfeld surfaces, reveals which atoms are the preferred partners of halogens in crystal packing contacts.

## 1. Introduction   

A crystal structure is determined by a combination of many forces where all the intermolecular interactions contribute. Interactions between molecules/functional groups are of an electrostatic or van der Waals nature. Analysing how molecules interact with their direct environment is an important step towards understanding crystal structure, packing formation and the relationship with thermodynamic properties.

There is a large body of literature investigating the role of halogen atoms in crystal interactions and crystal engineering. Organic Cl, Br and I atoms are considered to be hydrophobic. It has long been known that many intermolecular halogen *X*⋯*X* distances in molecular crystals can be significantly shorter (0.1–0.4 Å) than the sum of the accepted van der Waals radii (Sakurai *et al.*, 1963 ▶; Yamasaki, 1962 ▶; Nyburg, 1964 ▶). Halogen⋯halogen contacts have two preferred geometries described by the θ_1_ and θ_2_ C—*X*⋯*X* angles (Desiraju & Parthasarathy, 1989 ▶). Type I geometry is characterized by θ_1_ ≃ θ_2_, while in type II θ_1_ ≃ 180° and θ_2_ ≃ 90°. Notably, a Cambridge Structural Database (CSD, Version 1.15; Groom & Allen, 2014 ▶) statistical analysis of experimentally observed short *X*⋯*X* halogen contacts (*X* = F, Cl, Br or I) by Desiraju & Parthasarathy (1989 ▶) indicated that halogen⋯halogen interactions may be understood as specific weak attractive forces. *X*⋯*X* interactions are of special significance in the design of organic crystal structures, and examples of the utilization of halogen bonding in the production of functional materials are described by Ding *et al.* (2012 ▶). Halogens possess a global negative charge while maintaining an anisotropic electric potential around the atom. The polar electropositive region is known as a σ-hole. Halogen bonds have been described as directional electrostatically driven non-covalent interactions between the positive electro­static potential on the outer side of a halogen and an electrically negative site (such as the lone pair of a Lewis base or the π-electrons of an unsaturated system). Halogen bonds in protein–ligand complexes were recently reviewed by Sirimulla *et al.* (2013 ▶).

Analysis of intermolecular interactions using tools based on the Hirshfeld surface represents a major advance in enabling supramolecular chemists and crystal engineers to gain insight into crystal packing behaviour. The calculation and fingerprint representation of Hirshfeld surfaces is implemented in the program *CrystalExplorer* (Wolff *et al.*, 2012 ▶). The fast and easy visualization of close contacts using Hirshfeld surface analysis facilitates a quick summary of the intermolecular interactions. Importantly, the methodology can indicate which atom–atom contacts are the driving force for the crystal packing, as opposed to those that just happen to be placed next to each other. Hirshfeld surface analysis can be used in combination with the computation of the different contact enrichment ratios, described by Jelsch *et al.* (2014 ▶), to give a statistical picture of the intermolecular interactions in one or a series of crystal packings. The enrichment ratio is an indicator of the likelihood of chemical species to form intermolecular interactions with themselves and other species. It is a tool helpful for understanding the most important intermolecular interactions in a crystal structure, as it provides key information on the distribution of close contacts. In a previous study, several clear trends were found for contacts in crystals made of organic molecules containing a limited number of chemical species, namely CH, CHO, CHN, CHS and CHF.

In the current study, several families of halogenated mole­cules are retrieved from the CSD to investigate the partner preference of halogen atoms in crystal contacts. The enrichment ratios are determined for the intermolecular contacts, especially those involving halogen atoms. The preferred contact partners of F, Cl, Br and I atoms are identified, and differences in the behaviour of the four halogen types are highlighted.

## 2. Material and methods   

### 2.1. Hirshfeld surfaces   

Hirshfeld partitioning is an extension of the Hirshfeld stockholder concept (Hirshfeld, 1977 ▶), which divides the electron density of a molecule into continuous atomic fragments. The concept was generalized to extract continuous molecular fragments from electron-density distributions by defining a molecular weight function 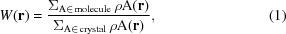
where ρ_A_(**r**) are spherically averaged atomic electron-density functions centred on the position of the atoms. The appropriate sums of the electron density of the atoms belong to the molecule and the crystal, respectively. Molecular properties can be obtained by integration over the weighted electron density and, using this scheme, molecular properties such as electrostatic moments have been reported (Moss & Coppens, 1980 ▶). The scheme is constructed by partitioning space into regions in which the electron-density sum over the spherical atoms of a molecule dominates the corresponding sum in the crystal (Spackman & Byrom, 1997 ▶; McKinnon *et al.*, 2004 ▶), *i.e. W*(**r**) > 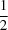.

The *CrystalExplorer* software (McKinnon *et al.*, 2004 ▶; Wolff *et al.*, 2012 ▶) is widely used by the community to display and analyse crystal packings and their resulting intermolecular interactions. A large range of properties can be visualized on the Hirshfeld surface, including the distance of atoms external, *d*_e_, and internal, *d*_i_, to the surface (Spackman & Byrom, 1997 ▶; Spackman & McKinnon, 2002 ▶), which can be shown in two-dimensional fingerprint plots. Normalized contact distances, *d*_norm_, have also been defined using van der Waals radii to highlight donors and acceptors and small and larger atoms equally on the surface. For H-atom positioning, *CrystalExplorer* uses average bond distances derived from neutron diffraction experiments (Spackman & Jayatilaka, 2009 ▶; Allen *et al.*, 2004 ▶).

### 2.2. Enrichment ratio   

The percentage *C*_*XY*_ of contacts on the Hirshfeld surface between two chemical elements *X* and *Y* in a crystal structure is determined by *CrystalExplorer*. The quantities can be used directly to calculate, by summation, the chemical content *S*_*X*_ of the Hirshfeld surface. The definition and calculation of contact enrichment ratios has been described previously (Jelsch *et al.*, 2014 ▶). Hence, the ratio of random contacts *R*_*XY*_ between two chemical elements *X* and *Y* is introduced. The *R*_*XY*_ values are defined as if all contact types *X*⋯*Y* in the crystal were equi-distributed between all chemical types and are obtained by probability products 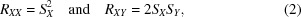
The factor 2 arises when reciprocal contacts *X*⋯*Y* and *Y*⋯*X* are both considered. Then, the enrichment ratio *E*_*XY*_ for a pair of elements (*X*, *Y*) is defined as the ratio between the proportion of actual contacts in the crystal and the theoretical proportion of equi-distributed random contacts 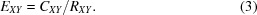
An enrichment ratio larger than unity reveals that a pair of elements has a high propensity to form contacts in a crystal structure, while pairs which tend to avoid contacts with each other should yield an *E* value lower than unity.

### 2.3. Selection of molecules   

Crystal structures were selected from the CSD based on their chemical composition and the aromatic/aliphatic character of the molecules. Only structures without disorder and with a single molecule in the asymmetric unit (*Z*′ = 1) were kept. The frequency of molecules with *Z*′ > 1 was small; for instance, among the CHCl compounds one aromatic and two aliphatic compounds were excluded due to *Z*′ = 2. At first, simple organic molecules that contain one type of halogen, such as CHCl, CHF, CHBr and CHI (aliphatic or aromatic halogenated hydrocarbons), were retrieved. Secondly, compounds containing two halogen atoms such as CHFCl and CHBrCl (aliphatic compounds) were selected to study the different types of *X*⋯*X* interactions. Thirdly, to analyse ‘halogen bonding’ with electronegative atoms such as O and N, molecules containing four different chemical elements such as CHFO, CHBrO and CHNCl were searched.

Aliphatic molecules were defined as devoid of double or triple bonds, although carbonyl and carboxylate groups were accepted in the oxygenated compounds. Aliphatic molecules have a large number of H atoms on their surface and therefore the C content of the Hirshfeld surface is small, as C atoms are involved in four covalent bonds with other atoms. As C is rare on the surface (*S*_C_ ≃ 0), contacts involving C atoms were not analysed in aliphatic molecules. The analysis of contacts was therefore simpler and the tendencies found are generally clearer in aliphatic compounds than in aromatics, as the number of variables describing the Hirshfeld surface content is smaller (*S*_H_ and *S*_*X*_, while *S*_C_ ≃ 0). Aromatic molecules were selected for having only aromatic groups, but double C=C bonds were also accepted. In this group, C is present in a significant proportion on the molecular surface and its interaction profile can be probed.

## 3. Results and discussion   

### 3.1. CHCl aliphatic compounds   

**Figure 1:**
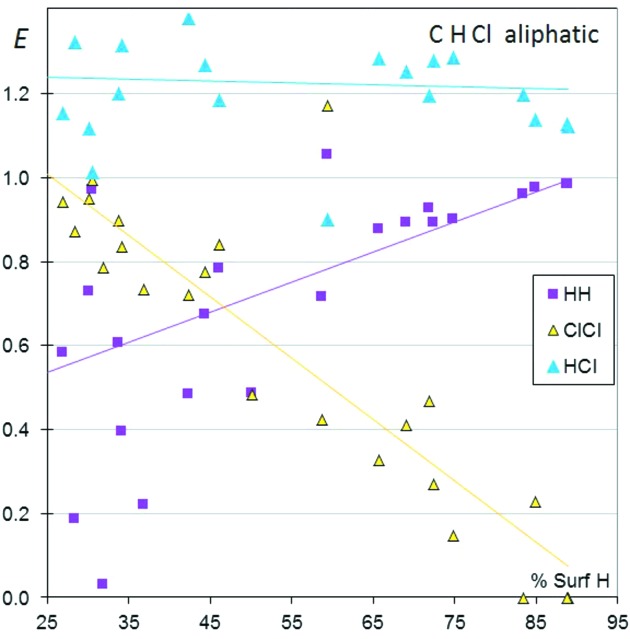
Contact enrichment ratios in crystals of CHCl aliphatic compounds as a function of the proportion of H on the Hirshfeld surface.

The crystal contact enrichment ratios for a series of aliphatic molecules containing only C, H and Cl are shown in Fig. 1 ▶ as a function of H content *S*_H_ on the surface. Globally, H⋯Cl contacts appear enriched, while the two H⋯H and Cl⋯Cl contacts are disfavoured. H⋯halogen interactions have been classified as very weak hydrogen bonds (Desiraju & Steiner, 2001 ▶). The hydrogen acceptor capabilities of ‘organic’ halogen, C—*X* (*X* = F, Cl, Br, I), with respect to hydrogen bonding have been considered controversial, and non-activated organic chlorine is generally deemed to be a poor acceptor. For example, Banerjee *et al.* (2004 ▶) reported the existence of intramolecular O—H⋯Cl—C interactions in several *gem*-alkynols. The *E*_HCl_ ratios are stable with varying *S*_H_, showing an average value of 1.22 and staying in the interval 0.9–1.5. In the context of CHCl molecules, this indicates that Cl is a better hydrogen acceptor than H. The H⋯Cl contacts are electrostatically favoured due to the complementary partial charges, δ^+^ of H and δ^−^ of Cl.

The *E*_ClCl_ values are generally lower than unity and they tend to decrease consistently with an increasing percentage of H at the molecular surface, from unity to zero. It should be noticed that the trend is the reverse with Cl content on the surface, as for aliphatic CHCl molecules. The *S*_H_ and *S*_Cl_ proportions are nearly complementary, with a sum close to unity. Cl⋯Cl contacts are disfavoured when the content of H is large on the molecular surface, due to competition with the more attractive H⋯Cl contacts. Incidentally, three molecules [C_15_H_26_Cl_2_, refcode CADINC01 (Wieczorek *et al.*, 1992 ▶); C_15_H_26_Cl_2_, REZHIO (Ourhriss *et al.*, 2007 ▶), and C_8_H_16_Cl_2_, XOQLAR (Karapetyan *et al.*, 2008 ▶)], which are very rich in H and poor in Cl, have no Cl⋯Cl contacts. The H⋯H contacts also generally display enrichment ratios lower than unity but increase globally with *S*_H_, as observed for other types of organic molecule such as CHN, CHO, CHS and CHF (Jelsch *et al.*, 2014 ▶). It should be recalled here that, when a chemical species is largely predominant (for instance *S*_H_ approaching 100%), the related enrichment ratio is statistically constrained to be close to unity. In contrast, the *E*_ClCl_ values decrease strongly with increasing *S*_H_ from values as high as unity to zero.

### 3.2. CHCl aromatic compounds   

**Figure 2:**
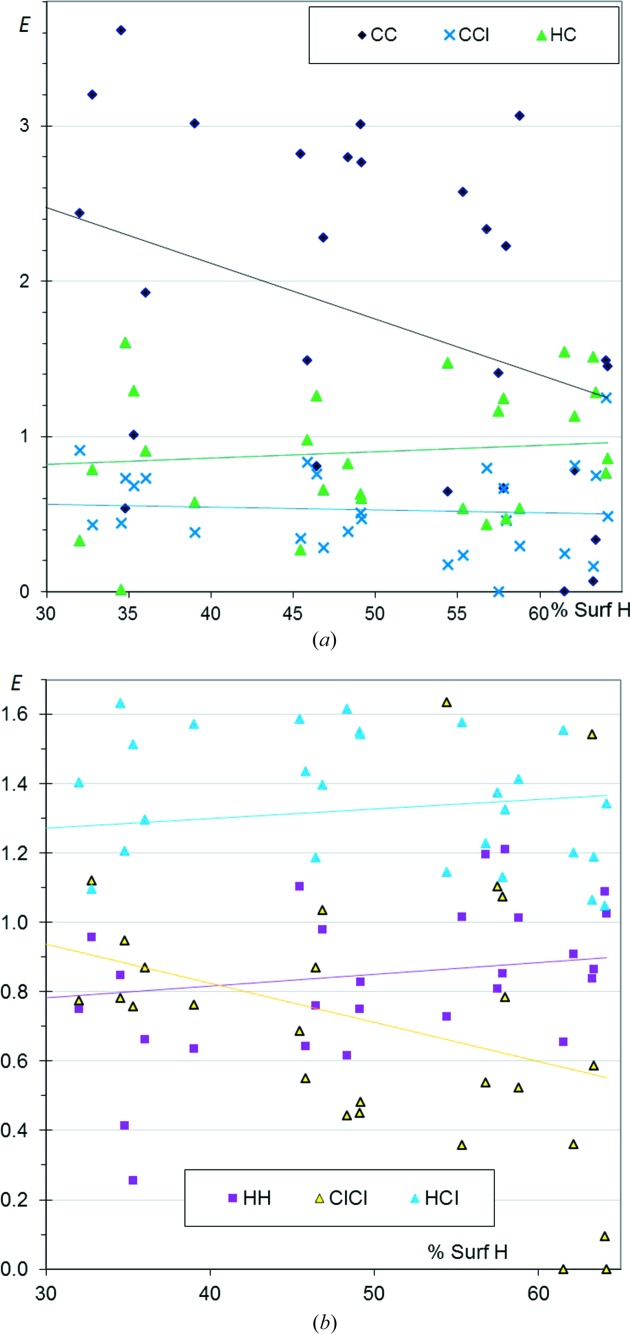
Contact enrichment ratios in crystals of CHCl aromatic compounds. (*a*) Contacts involving C; (*b*) other contacts. In the case of a poor correlation (for example *R* ^2^ = 0.04 for *E*_HCl_), the best fit lines lose some of their statistical meaning but still indicate the average trends of the ordinate axis variable. Therefore, the average values of different ordinate variables can still be compared through the best fit lines.

By decoding the (*S*_H_, *E*_*XY*_) scatterplots plots (Fig. 2 ▶), the specific types of contact associated with the selected CHCl aromatic compounds can be characterized. The C⋯C contacts show a wide range of enrichment from 0 to 3.6 (Fig. 2 ▶ *a*) and are, on average, the most enriched ones. Values as high as 3.5 have already been observed in several other families of aromatic molecules (CHO, CHN, CHS, CHF; Jelsch *et al.*, 2014 ▶). These high *E*_CC_ values highlight the importance of π–π stacking in chlorinated aromatic compounds. π–π stacking is favoured in heterocyclic compounds as the molecules have the ability to find orientations which are complementary from an electrostatic perspective (Salonen *et al.*, 2011 ▶).

The C⋯Cl contacts display *E* ratios between 0.0 and 1.2 and show the lowest enrichment of all the contact types, with an average value below 0.6. C⋯H contacts are, on average, only slightly disfavoured, with 〈*E*_CH_〉 = 0.9 and some compounds having *E*_CH_ reaching 1.6. The two types of contact show stable values as a function of the proportion of H on the molecular surface.

Globally, similar trends are found for contacts not involving C in aromatic and aliphatic compounds. In the case of aliphatic molecules, the points are closer to the fitted line, as the proportion of C on the surface is a non-intervening variable (*S*_C_ close to zero). However, the *E*_HCl_ ratio is larger, on average, for aromatic molecules (1.3 *versus* 1.2), which may be related to the stronger acidity of H on aromatic rings or double bonds compared with aliphatics. The results for CHCl aromatic compounds suggest that π–π stacking and H⋯Cl interactions are the driving forces in molecular arrangement and crystal packing formation.

The peculiar case of hexachlorobenzene, C_6_Cl_6_, has been analysed by Bui *et al.* (2009 ▶), who found that triplets of Cl atoms form triangles of interactions. This type of halogen⋯halogen interaction can be explained by the atomic charge density. Organic Cl tends to be slightly negatively charged through an equatorial torus of electron accumulation, while there is an electron depletion towards the polar C–Cl direction. In hexachlorobenzene, with *S*_Cl_ = 83%, the enrichment values are *E*_CC_ = 3.6, *E*_ClCl_ = 1.1 and *E*_CCl_ = 0.5. In this particular case of a CHCl aromatic molecule where H is absent, Cl prefers to interact with itself rather than with C. The compounds C_6_Br_6_ and C_6_I_6_ show the same crystal packing and interactions, as the charge densities of organic Br and I atoms show similar features.

### 3.3. CHF aliphatic compounds   

**Figure 3:**
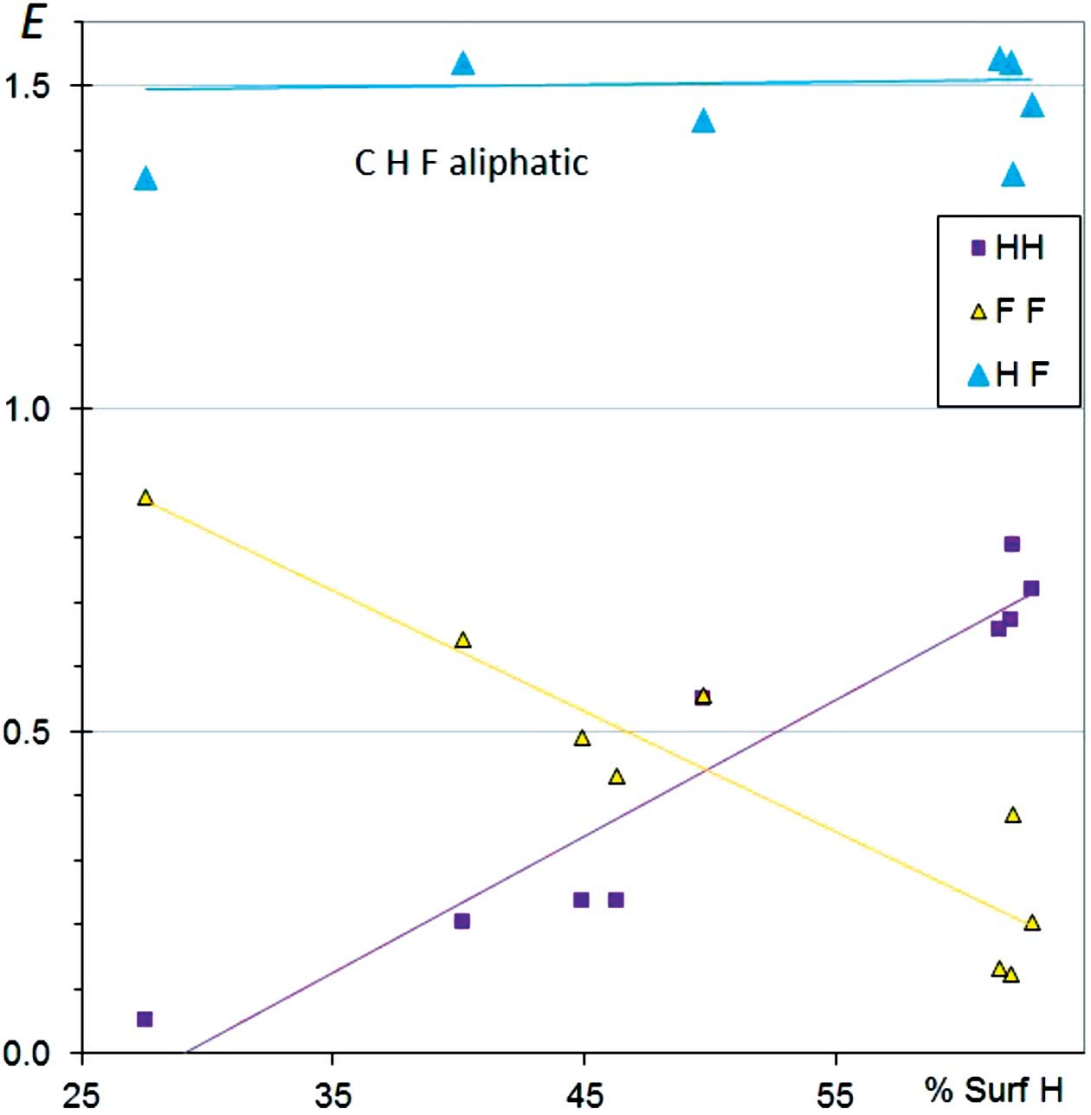
Contact enrichment ratios in crystals of CHF aliphatic compounds as a function of the proportion of H on the Hirshfeld surface.

Fluorine behaves differently from the other halogens because of its small size, weak polarizability and higher electro­negativity and electron-withdrawing effect. The charge density of organic fluorine still displays the same shape as other halogens, with an electronegative torus and an electropositive region along the C–F axis (Chopra & Row, 2011 ▶). This anisotropic distribution of the electron density around organic fluorine and other halogen atoms is also referred as ‘polar flattening’ (Nyburg, 1979 ▶). Fig. 3 ▶ depicts the distribution of contacts in aliphatic fluorinated compounds. Nine molecules of this type were selected from the CSD. Fluorine prefers H⋯F interactions rather than F⋯F contacts, due to the dipolar character of the H⋯F interaction. Globally, the tendencies found are similar to those for chlorinated aliphatic compounds (Fig. 1 ▶). However, H⋯F contacts are significantly more favoured than H⋯Cl (〈*E*_H*X*_〉 = 1.4 *versus* 1.2), presumably due to the stronger electron-withdrawing effect of F.

### 3.4. CHF aromatic compounds   

**Figure 4:**
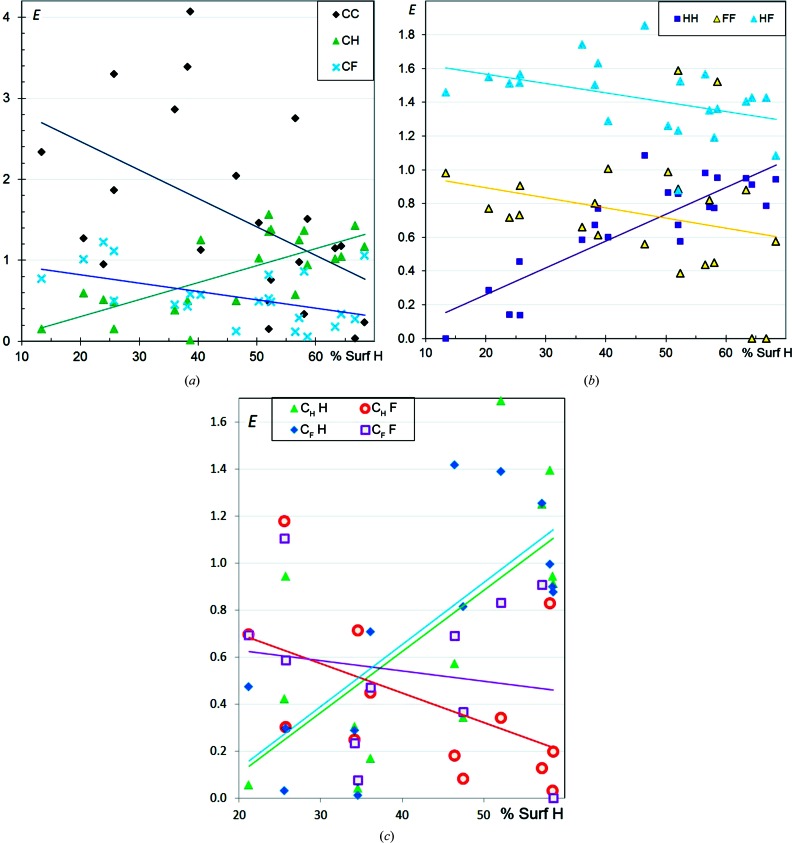
Contact enrichment ratios in crystals of CHF aromatic compounds. (*a*) Contacts involving C. (*b*) Other contacts. (*c*) C⋯H and C⋯F interactions, with a distinction between C atoms bound to F (C_F_) and bound only to H and C atoms (C_H_). In part (*c*), *E*_*XY*_ points derived from *R*_*xy*_ values smaller than 1.5% were discarded as they correspond to ratios of very small numbers.

In CHF aromatic compounds, the enrichment of the C⋯C contacts can take a wide range of values between 0 and 4 (Fig. 4 ▶ *a*) and the global trend is that *E*_CC_ decreases strongly with increasing *S*_H_. On the other hand, *E*_CH_ values increase steadily from 0 to 1.5 when the H content on the molecular surface increases. This behaviour is similar to that found in CHN and CHO aromatic compounds (Jelsch *et al.*, 2014 ▶). C—H⋯π interactions are favoured when H atoms are highly available, but are replaced by some C⋯C contacts, which correspond to parallel π-stacking, when H atoms are less abundant. Also, the behaviour of fluorinated aromatic compounds with a high H content tends to resemble that of pure CH aromatic compounds, where H⋯C contacts are preferred to C⋯C contacts. The *E*_CF_ values are generally lower than unity and decrease regularly as the H content becomes high. The CHCl aromatic compounds display a different behaviour, with stable *E* values for the C⋯H and C⋯Cl interactions.

F⋯F interactions are slightly more disfavoured than Cl⋯Cl contacts (Fig. 2 ▶ *b*), the trend for their enrichment ratio to decrease with increasing *S*_H_ being similar. The *E*_FF_ values tend, on average, to decrease from 0.8 to 0.2 with increasing *S*_H_. Hathwar *et al.* (2014 ▶) found experimental evidence for the polarization of the electron density on the F atom, with the formation of an electron-deficient region along the C—F axis which can interact favourably with the electronegative torus of a neighbouring F. However, the electropositive region has a reduced size in F compared with the larger halogen atoms. The comparison of *E*_HF_ and *E*_FF_ in Fig. 2 ▶(*b*) confirms previous findings that the C—F group prefers to form C—H⋯F interactions rather than F⋯F contacts (Thalladi *et al.*, 1998 ▶).

On the other hand, *E*_HH_ increases strongly from zero to unity as the H content increases. Supplementary Fig. S1 also shows that *E*_HH_ decreases strongly with increasing F content, while both *E*_FF_ and *E*_HF_ increase slightly with increasing *S*_F_.

It can be concluded that, in aromatic CHF molecules with a high F content or low H content, H atoms tend to form H⋯F interactions while C atoms are mostly involved in C⋯C contacts (π–π parallel stacking). On the other hand, CHF molecules with low *S*_F_ or high *S*_H_ have more enriched C⋯H interactions. Highly fluorinated compounds display many C⋯C contacts, while C⋯F interactions which are part of the so-called ‘halogen bonding’ become more abundant (*E*_FF_ close to unity).

CHCl aromatics show different behaviour, with *E*_HH_ more stable around an average value of 0.8. Another difference from chlorinated compounds is that *E*_HF_ tends to decrease slightly from 1.6 to 1.3 with increasing *S*_H_, unlike *E*_HCl_ which remains more stable around the average value of 1.3.

The analysis was further refined by distinguishing C atoms bound to F atoms (C_F_), which are therefore affected by its electron-withdrawing effect, from other C atoms (C_H_). Fig. 4 ▶(*c*) shows no significant difference in the behaviour of C_F_⋯H and C_H_⋯H interactions. On the other hand, C_F_⋯F contacts appear slightly less disfavoured than C_H_⋯F for molecules rich in H (or poor in F). The enrichment ratios for contacts between the two C-atom types, shown in Fig. S2, take a wide range of values; C_F_⋯C_H_ interactions seem more enriched than C_H_⋯C_H_ contacts for molecules poor in H.

### 3.5. CHBr aliphatic compounds   

**Figure 5:**
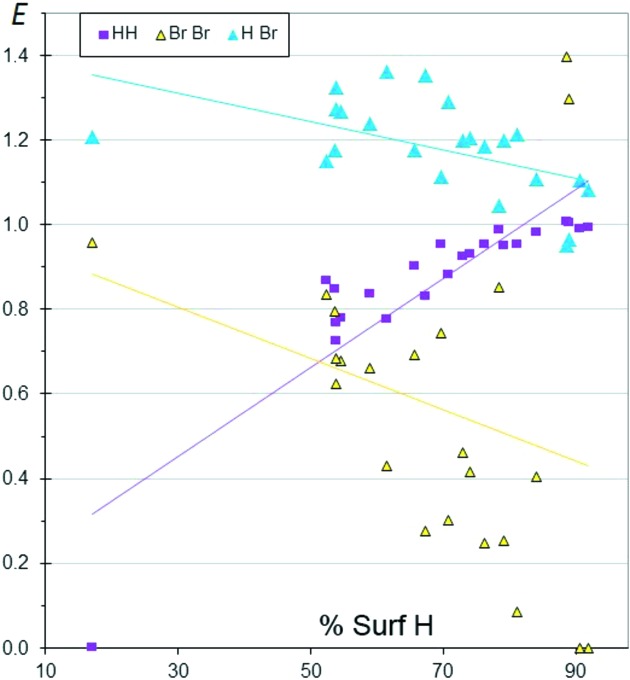
Contact enrichment ratios in crystals of CHBr aliphatic compounds as a function of the proportion of H on the Hirshfeld surface.

The crystal contact propensities in CHBr aliphatic mol­ecules are similar to their CHCl counterparts (Fig. 5 ▶). Notably, the *E*_HBr_ and *E*_HCl_ ratios have similar average values around 1.2 but are smaller than *E*_HF_. The main discrepancy is that *E*_HBr_ decreases slightly with increasing *S*_H_. One compound which is poor in H, CHBr_3_, has no H⋯H contact (*E*_HH_ = 0). This molecule crystallizes in the polar space group *P*6_3_, with all molecules aligned along one direction, so that the H atoms do not come close to each other. Presumably, aligning the dipole moments of the CHBr_3_ molecules is an important driving force for this packing formation. On the other side of the scatterplot, two molecules [C_15_H_23_Br, BCYLON10 (Thierry & Weiss, 1972 ▶), and C_16_H_19_Br, BHPCHD10 (Osawa *et al.*, 1980 ▶)] which are poor in Br have *E*_BrBr_ = 0. These two molecules have an ellipsoid shape not far from a sphere (like CHBr_3_). The unit cell of BHPCHD10 has two relatively short axes (*a* = 6.5 and *b* = 8.1 Å) compared with the third, long, axis (*c* = 23.0 Å), with the molecules forming layers parallel to the (001) planes, where molecules are related by translations and the dipole moments are aligned. The same can be said of the packing of BCYLON10, which has unit-cell parameters *a* = 20.5, *b* = 6.9 and *c* = 9.2 Å. ‘Spherically shaped’ CHBr molecules with a unique H or Br atom have an electric moment which is mostly dipolar, so such molecules may tend to form crystal packings with significant dipole alignment.

### 3.6. CHBr aromatic compounds   

**Figure 6:**
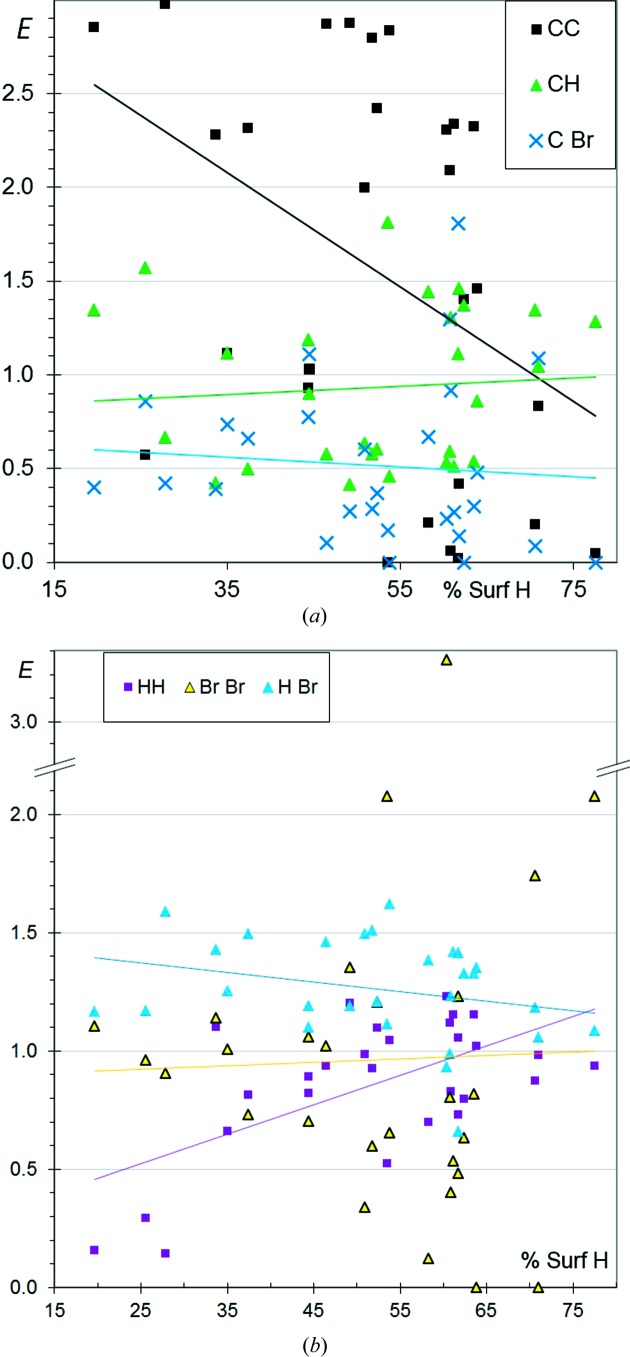
Contact enrichment ratios in crystals of CHBr aromatic compounds as a function of the proportion of H on the Hirshfeld surface. (*a*) Contacts involving C; (*b*) other contacts.

The selected CHBr aromatic compounds contain 10–34% C, 19–77% H and 6–63% Br on the molecular surface. The enrichment trends (Fig. 6 ▶) are similar to those of CHCl (Fig. 2 ▶) and, to a lesser degree, to those of CHF aromatic compounds (Fig. 4 ▶). The H⋯halogen contacts are slightly more enriched, on average, in chlorinated and fluorinated compounds compared with CHBr aromatic compounds. The *E*_HBr_ ratio decreases slightly with increasing *S*_H_ and increases slightly with increasing *S*_Br_ (Fig. S3). With an average *E*_BrBr_ ratio of 0.9, the Br⋯Br contacts appear to be less disfavoured than F⋯F and Cl⋯Cl contacts. The halogen⋯halogen interaction is favoured by the electrostatic anisotropy of halogen atoms, but in the case of Br the higher polarizability of this atom may also be a contributing factor. Incidentally, three molecules with high H content show Br⋯Br contact enrichment values larger than 2. This is attributed to molecules with a limited Br content (7 < *S*_Br_ < 17%), and then the presence of one or two Br⋯Br contacts in the crystal packing can result in a mathematically large *E*_BrBr_ ratio.

**Figure 7:**
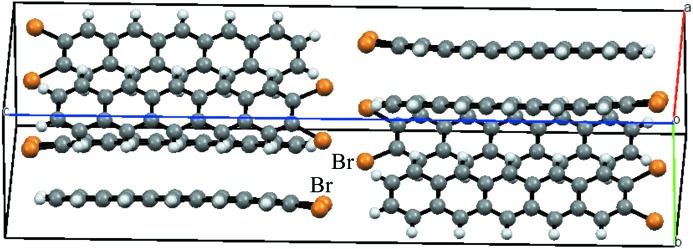
The crystal packing of the compound C_22_H_12_Br_2_ (OKANOE; Okamoto *et al.*, 2010 ▶) with a particularly high *E*_BrBr_ ratio. Two thirds of the unit cell are shown (space group *Pbca*, *Z* = 8).

Fig. 7 ▶ shows the crystal packing of an outlier molecule in the Fig. 6 ▶(*b*) scatterplot. The ratio *E*_BrBr_ = 2.1 is high while the content of Br on the surface is only moderately small (*S*_Br_ = 17%). This crystal packing is actually characterized by three other outlier values: *E*_CC_ = 0, a high *E*_CH_ = 1.8 and a low *E*_HH_ = 0.5. This compound is an elongated CH aromatic molecule with two Br atoms at one extremity. The driving force in this crystal packing formation seems to be the same as that observed for CH aromatics (Jelsch *et al.*, 2014 ▶): the establishment of many electrostatically favourable C^δ−^⋯H^δ+^ interactions and the avoidance of H⋯H and C⋯C contacts. As a result of this packing arrangement, the Br atoms at the molecular extremity interact with H and Br atoms of adjacent molecules. The other two outliers (*E*_BrBr_ > 2) are molecules with a unique Br atom forming Br⋯Br interactions through crystallographic symmetry in the Type I geometry (C—H⋯Br = θ_1_ = θ_2_). One of the two molecules comprises a planar system of four adjacent rings, while the other is made up of three aromatic rings pointing in three directions.

### 3.7. CHI aliphatic compounds   

**Figure 8:**
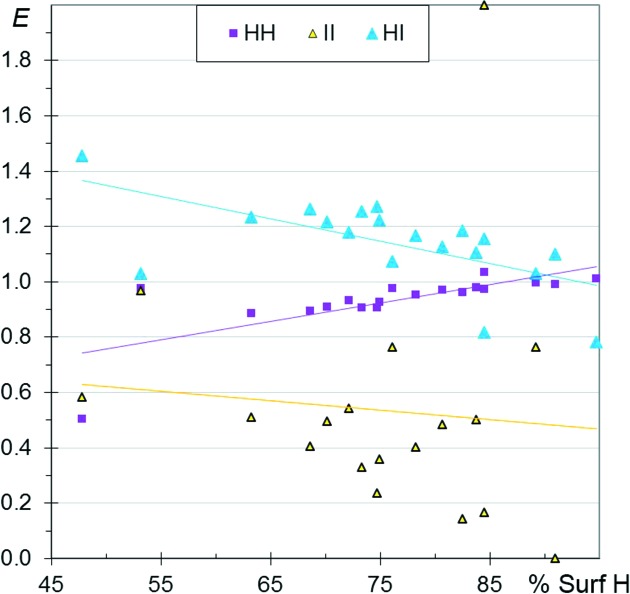
Contact enrichment ratios in crystals of CHI aliphatic compounds as a function of the proportion of H on the Hirshfeld surface.

**Figure 9:**
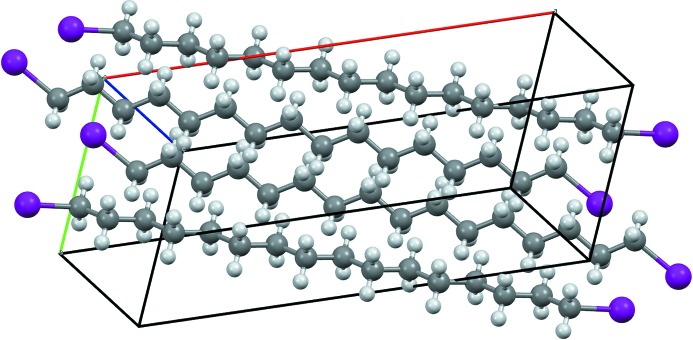
The crystal packing of the aliphatic compound 1,16-diiodohexadecane with a particularly high *E*_II_ ratio. The four molecules in the unit cell are shown (space group *P*2_1_/*c*).

The packing contacts in CHI aliphatic molecules (Fig. 8 ▶) show the same enrichment profile as the corresponding CHBr and CHCl compounds, with H⋯I being a favoured inter­action. One molecule can be considered as an outlier, with an I⋯I contact enrichment reaching 2.0 and its *E*_HI_ = 0.8 value lower than the average trend. This compound, 1,16-diiodohexadecane (C_16_H_32_I_2_), contains 85% H and 15% I on the molecular surface. The peculiar crystal enrichment contacts of this molecule are related to its particular shape: the compound forms a long linear chain with I atoms at both extremities. The crystal packing (Fig. 9 ▶) shows parallel chains interacting with each other laterally through H⋯H contacts which represent 74% of all interactions. The I atoms are located close to planes parallel to (100) and the molecules form tail-to-tail inter­actions, mainly through I⋯I contacts. The high proportion of I⋯I contacts in this case occurs through crystallographic symmetry and may be a secondary consequence of the crystal packing arrangement of the long molecular chain.

### 3.8. CHI aromatic compounds   

**Figure 10:**
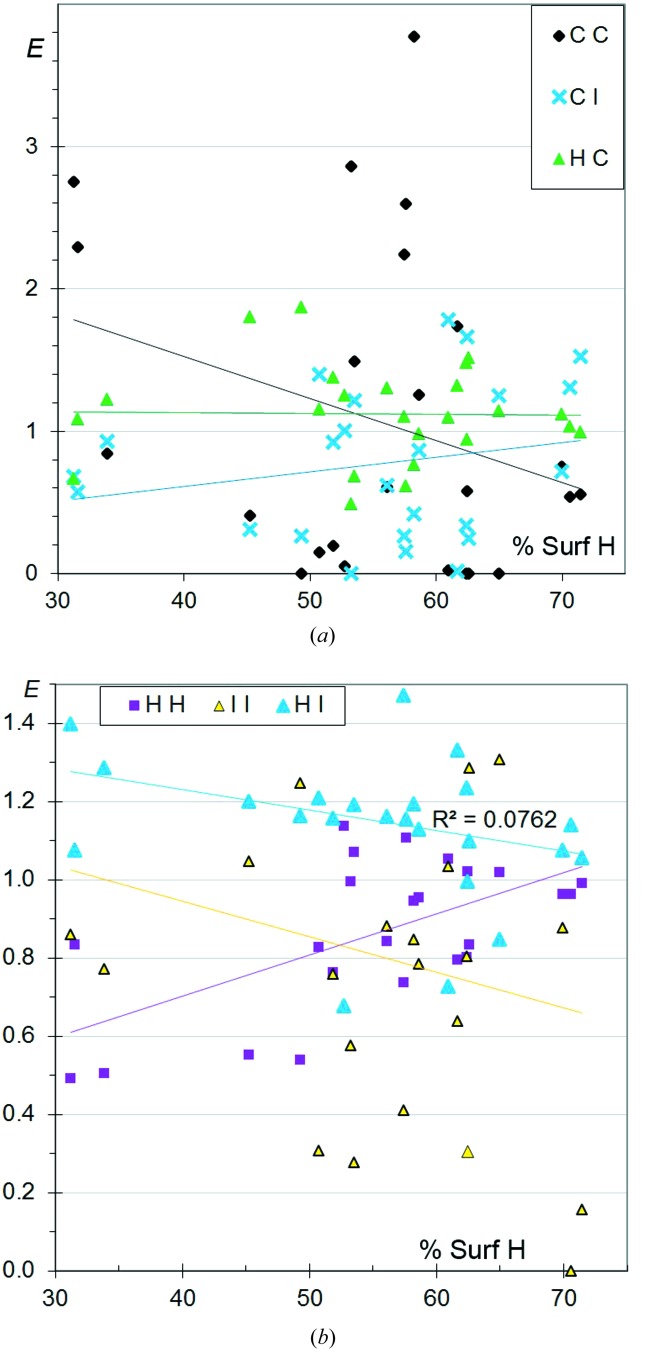
Contact enrichment ratios in crystals of CHI aromatic compounds. (*a*) Contacts involving C; (*b*) other contacts.

In CHI aromatic compounds (Fig. 10 ▶ *a*), the contact likelihoods show similar trends to the other three halogen species. The graph of *E* ratios (Fig. 10 ▶ *a*) shows that the average propensity of C⋯I and H⋯C interactions remains stable as a function of *S*_H_ values. All three types of contact involving C show a large variability among the molecules. Notably, *E*_CC_ has values ranging from 0 to 3.4 for molecules with intermediate H content on the surface (around 50 to 60%). The enrichment ratios are essentially independent of the proportion of C on the molecular surface (Fig. S4). The C⋯I contacts, although globally disfavoured, show *E* values between 0 and 1.8, and they are the most favoured interactions for molecules rich in H or C and poor in I.

### 3.9. Comparison of the aliphatic halogenated compounds   

**Figure 11:**
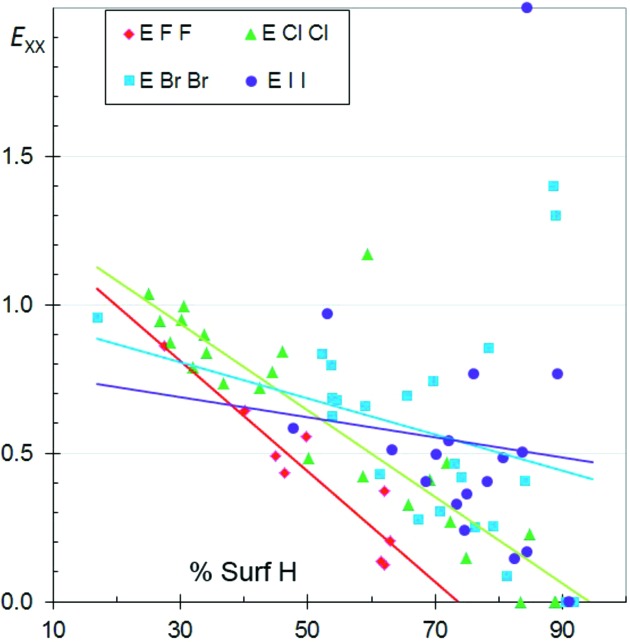
Comparison of halogen⋯halogen contact enrichment ratios in crystals of aliphatic compounds.

The enrichment ratios of the different halogen⋯halogen (*X* = F, Cl, Br, I) contacts in aliphatic CH*X* compounds as a function *S*_H_ values are shown together in Fig. 11 ▶. The *E*_*XX*_ values are generally smaller than unity, indicating that halogen⋯halogen interactions are not the most favoured contacts in aliphatic CH*X* compounds. Globally, all the *E*_*XX*_ values decrease as H becomes more abundant on the molecular surface, as mentioned in previous sections. The *E*_FF_ ratios, followed by the *E*_ClCl_ ratios, tend to have the smallest values, independent of the H content on the Hirshfeld surface. F⋯F contacts are the most unfavourable among *X*⋯*X* contacts, while Br⋯Br and I⋯I contacts are less unfavourable, presumably because the heavier halogens are less electronegative and more polarisable.

The fitted curves of *E*_*XX*_ as a function of H content on the surface have the strongest negative slope for F, followed by Cl. This can be connected to the fact that the H⋯F and H⋯Cl contacts are the most attractive (as also seen in Fig. 3 ▶) while H⋯Br and H⋯I are the least attractive, in relation to the stronger electronegativity of the smaller halogens. The *E*_H⋯*X*_ ratios are compared in the same scatterplot for the different families of CH*X* compounds in Fig. S5. The *E*_H⋯F_ ratios appear to be larger than for the other three halogen H⋯*X* contacts for both aromatic and aliphatic compounds.

### 3.10. CHCl oxygenated aliphatic compounds   

**Figure 12:**
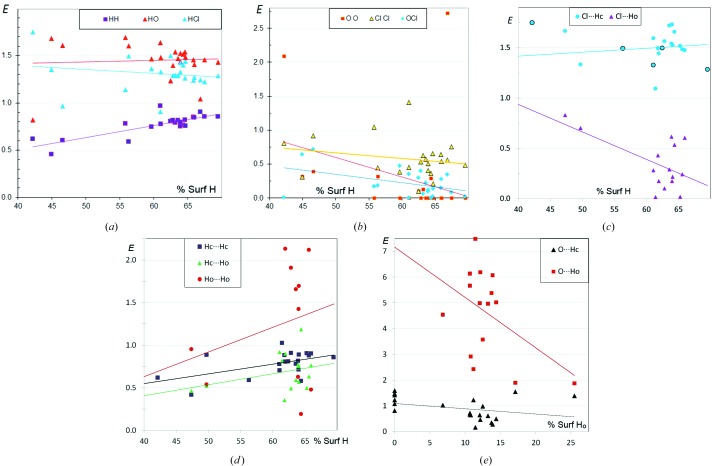
Contact enrichment ratios in crystals of CHClO aliphatic compounds. (*a*) Contacts involving H, (*b*) other contacts, (*c*) distinction between Cl⋯H_C_ and Cl⋯H_O_ contacts, (*d*) distinction between H_C_⋯H_C_, H_C_⋯H_O_ and H_O_⋯H_O_ contacts, and (*e*) distinction between O⋯H_C_ and O⋯H_O_ contacts. Points are discarded when *R*_*XY*_ < 1.5%.

The scatterplots in Fig. 12 ▶ compare the contact propensities of O and Cl in a series of CHOCl aliphatic compounds. The O atoms belong to a hydroxyl, carbonyl, carboxylic acid or ether group. The scatterplots are drawn as a function of *S*_O_, and the same data as a function of *S*_Cl_ are shown in Fig. S6. The *E*_OO_ values are generally very small, often equal to zero, but there are some exceptions, with two compounds rich in O⋯O contacts [C_4_H_4_Cl_2_O_2_, DISZIO (Ducourant *et al.*, 1986 ▶), and C_6_H_11_Cl_3_O_2_, CUGPUQ (Nilewski *et al.*, 2009 ▶)]. The *E*_OCl_ ratios are also small and below 0.7. However, among the different types of halogen⋯O contact it has been reported that halogen⋯O(nitro) interactions are attractive and often present in crystal structures containing both chemical groups (Allen *et al.*, 1997 ▶). The nitro O atom is indeed less electronegative than the hydroxyl or carbonyl O atoms found in the present sample. Compared with Cl⋯O and O⋯O inter­actions, Cl⋯Cl contacts are slightly less disfavoured.

The average enrichment ratios *E*_HCl_ = 1.3 and *E*_HO_ = 1.45 (Fig. 12 ▶ *b*) confirm that O is a stronger hydrogen-bond acceptor than Cl. The *E*_HO_ value is globally stable with the three variables *S*_H_, *S*_O_ and *S*_Cl_ (Fig. S6). The *E*_HCl_ value is, on average, stable with varying *S*_O_, and decreases/increases very slightly with increasing *S*_H_ and *S*_Cl_, respectively. The *E*_HH_ ratio is always lower than unity. It is stable with varying *S*_Cl_ (the proportion of the weak hydrogen-bond acceptor) but clearly diminishes with increasing *S*_O_.

In Fig. 12 ▶(*c*), the H atoms bound to C and O (H_C_ and H_O_) are differentiated. The Cl atoms display a much higher contact affinity with H_C_ atoms than with H_O_. The Cl⋯H_C_ contacts show enrichment ratios of around 1.5 which are stable with varying *S*_H_. Conversely, Cl⋯H_O_ interactions turn out to have systematically impoverished occurrences, presumably as the more electropositive H_O_ atoms prefer to form hydrogen bonds with O atoms. This is confirmed by the high *E*_OH_O__ ratios, which are generally larger than 2 and even as high as 7. On the other hand, O⋯H_C_ contacts show a lower average enrichment of around unity. The highest *E*_OH_C__ enrichments occur for compounds devoid of H_O_ atoms, for instance ketones. Globally, for both types of O⋯H interaction, the *E* ratios tend to decrease with increasing *S*_H_O__.

Concerning H⋯H contacts, H_C_⋯H_C_ is slightly less dis­favoured than H_C_⋯H_O_ (Fig. 12 ▶ *d*). On the other hand, H_O_⋯H_O_ interactions show a large range of enrichment ratios between 0 and 2. In some compounds, high *E*_H_O__ _H_O__ values might be a secondary effect of the very frequent O⋯H—O hydrogen bonding (Fig. 12 ▶ *e*), due to the proximity of O and H_O_ atoms.

### 3.11. CHFO aliphatic compounds   

**Figure 13:**
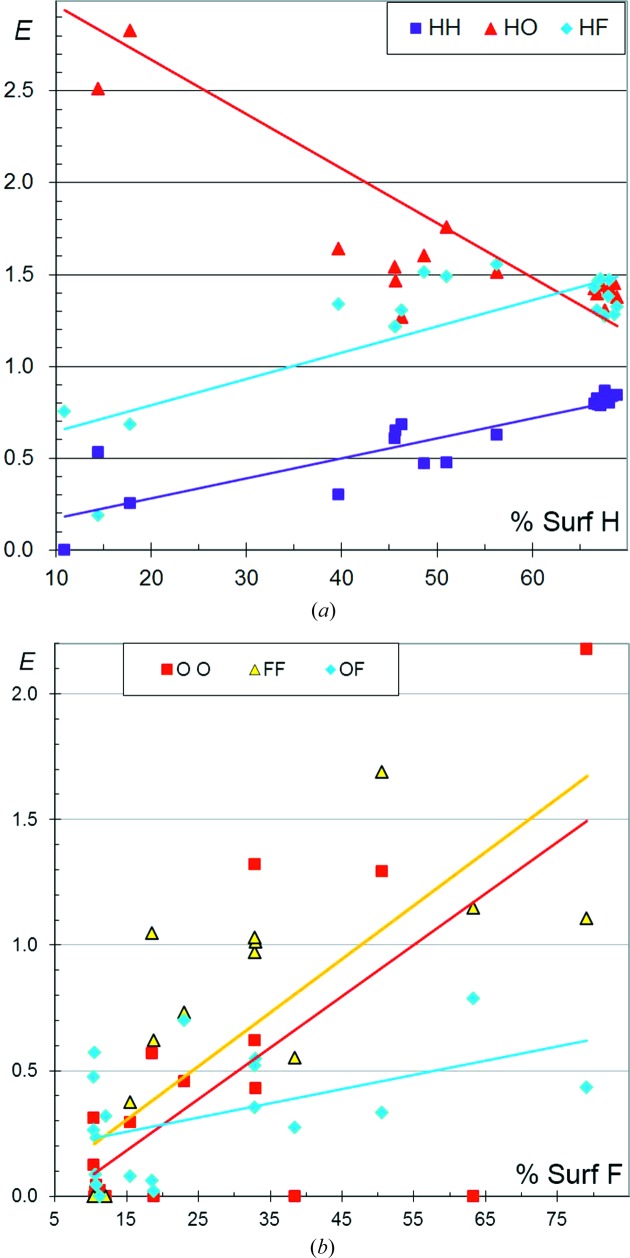
Contact enrichment ratios in crystals of CHFO aliphatic compounds. (*a*) Contacts involving H; (*b*) other contacts.

The role of organic fluorine in crystal packing and engineering was reviewed by Chopra & Guru Row (2011 ▶). Interactions involving two electronegative atoms among the F and O species are on average disfavoured, notably O⋯F contacts (Fig. 13 ▶ *b*). Globally, the three *E* ratios increase strongly with increasing *S*_F_ and slightly with increasing *S*_O_ (Fig. S7). Concomitantly, O⋯F, O⋯O and F⋯F contacts are strongly disfavoured when H is abundant in the molecule, due to competition with more favourable H⋯F and H⋯O inter­actions. At low H content, the O⋯F interactions remain the most avoided contacts (Fig. S7).

The trends found for contacts involving H in CHFO aromatic molecules (Fig. 13 ▶ *a*) show some differences with those observed in their chlorinated counterparts. The *E*_HO_ and *E*_HF_ values strongly decrease/increase, respectively, as a function of H molecular content. For molecules rich in H, where all F and O atoms are hydrogen-bonded, the *E*_HO_ and *E*_HF_ enrichment ratios are both close to 1.4. When H is rare, H⋯O hydrogen bonds are formed to the detriment of the weaker H⋯F contacts. Therefore, H⋯F interactions involving organic F are rarer in the presence of strong hydrogen bonds.

Indeed, H⋯F contacts appear to be generally favoured in small-molecule crystal structures. In medicinal chemistry, the formation of intermolecular O—H⋯F—C hydrogen bridges was assumed to be important in binding fluorinated compounds to enzyme active sites (Chopra & Guru Row, 2011 ▶). For example, the compound 2,2,3,3-tetrafluorobutane-1,4-diol has two O-bound and four C-bound H atoms. Both *E*_HF_ = 1.5 and *E*_HO_ = 1.75 are larger than unity, but there is a preferential formation of O—H⋯O and C—H⋯F inter­actions within the crystal structure (Fig. S8). The strong hydrogen-bond acceptors (O) and donors (H_O_) associate, while the weak acceptors (halogens) and donors (H_C_) interact secondarily, as already observed in CHClO compounds in Figs. 12 ▶(*c*)–12 ▶(*e*).

### 3.12. CHBrO aliphatic compounds   

**Figure 14:**
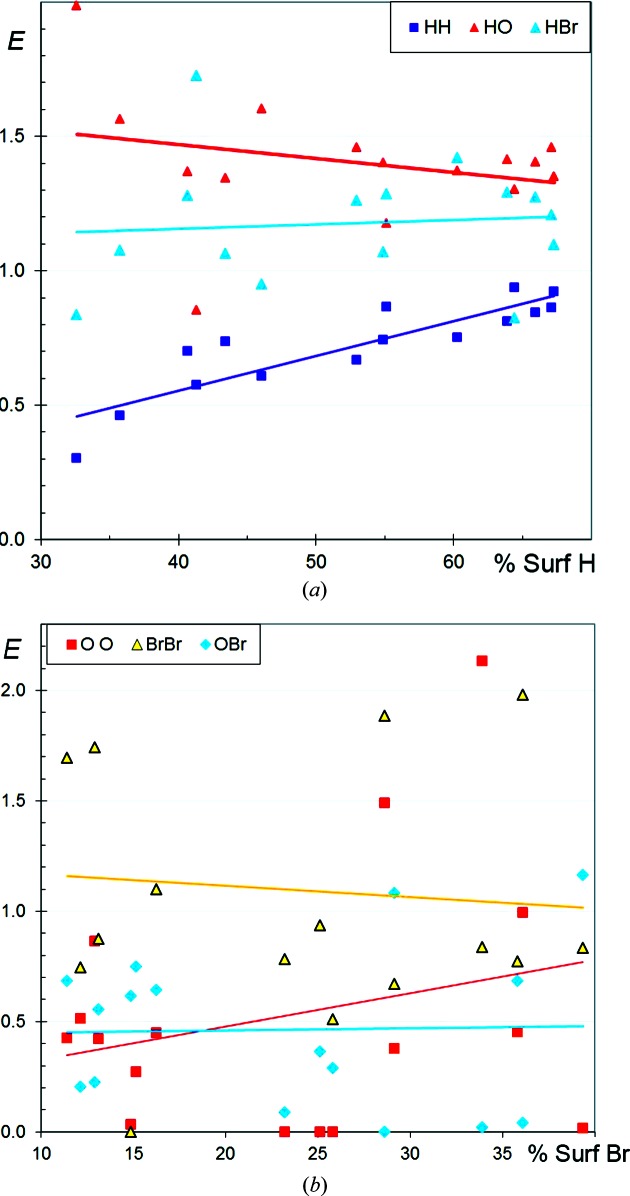
Contact enrichment ratios in crystals of CHBrO aliphatic compounds. (*a*) Contacts involving H; (*b*) other contacts.

O⋯O and O⋯Br contacts are both generally disfavoured in the 13 CHOBr aliphatic compounds (Fig. 14 ▶ *b*). The O⋯O contacts are totally absent for five compounds but are very enriched (*E*_BrBr_ > 1.5) for two of them. The Br⋯Br contacts show disparate enrichments between 0.0 and 2.2, with two clusters around 0.8 and 1.8. The Br⋯Br contacts are less disfavoured in oxygenated CHBrO compounds than in CHBr molecules. Compared with CHClO compounds, halogen⋯halogen contacts also more favoured here. Globally, all contacts involving only the electronegative atoms O and Br have a greater propensity to occur when O is abundant or H is rare on the molecular surface (Fig. S9).

In Fig. 14 ▶(*a*), the *E*_HO_ values are higher than *E*_HBr_, in accordance with O being a stronger hydrogen-bond acceptor than Br. The *E*_HBr_ ratios are also smaller on average than *E*_HCl_ (1.16 *versus* 1.3), their counterpart in the CHClO aliphatic compounds in Fig. 12 ▶(*a*), indicating that Br is a weaker hydrogen-bond acceptor. The *E*_HBr_ and *E*_HO_ values are both stable with varying Br content, but when the O proportion in the molecule increases, *E*_HBr_ tends to decrease and *E*_HO_ to increase. These trends further confirm the stronger hydrogen-bond acceptor character of O.

### 3.13. CHNCl aromatic compounds   

**Figure 15:**
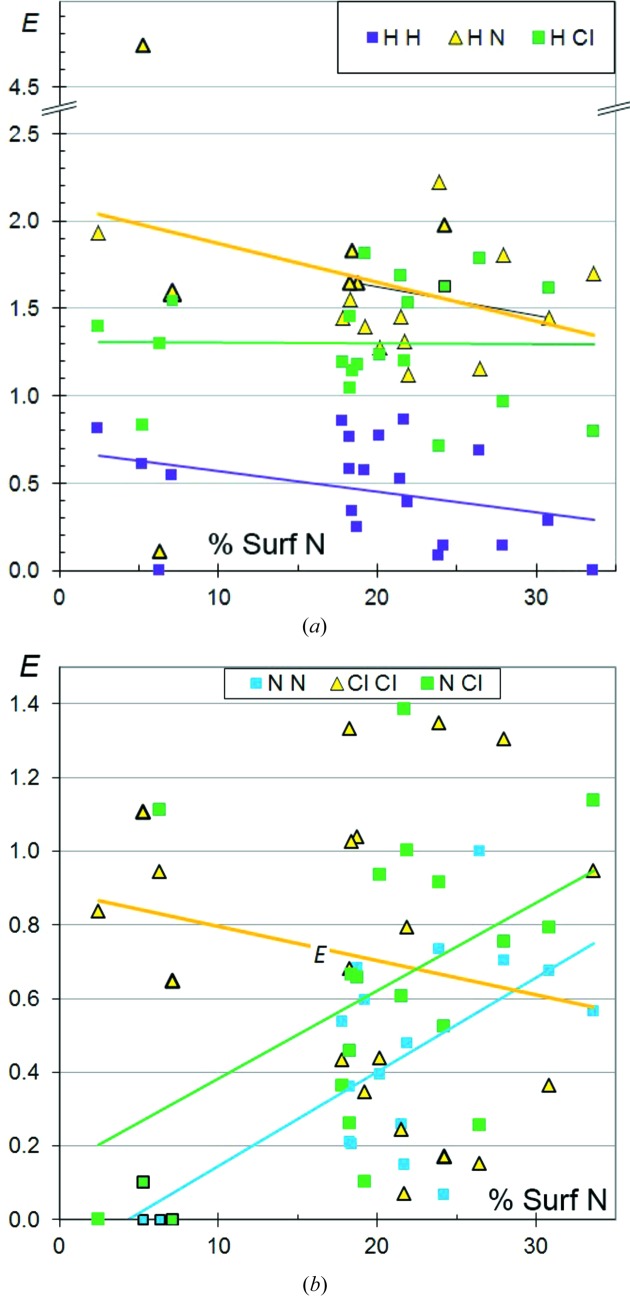
Contact enrichment ratios in crystals of CHNCl aliphatic compounds as a function of N proportion on the Hirshfeld surface. (*a*) Contacts involving H, (*b*) other contacts. The *E*_HN_ points are highlighted by thick dark borders when all the N atom(s) in the compound have an electron lone pair.

To analyse the propensity of halogen⋯N contacts to form, aromatic CHNCl compounds were considered. Aliphatic molecules were not considered because (amine) N atoms generally form four covalent bonds and do not contribute much to the molecular surface content. H⋯N contacts seem to be more favoured in chlorinated CHN aromatic compounds, with 〈*E*_HN_〉 = 1.7 (Fig. 15 ▶), compared with 〈*E*_HN_〉 of only 1.2 for CHN aromatic molecules (Jelsch *et al.*, 2014 ▶). The *E*_HN_ points are highlighted in Fig. 15 ▶(*a*) when all the N atoms in the compound are hydrogen-bond acceptors (N atom with two covalent bonds, not bound to H); the corresponding *E*_HN_ values are generally larger than 1.5. One compound [C_6_HNCl_6_, 2,3,6-trichloro-5-(trichloromethyl)pyridine, QEDCAF (Zhu *et al.*, 2012 ▶)] is an outlier as its *E*_HN_ value is close to zero, since the unique C-bound H atom is a weak hydrogen-bond donor and interacts preferentially with Cl atoms. H⋯N contacts are generally more enriched than H⋯Cl, which recalls the results with H⋯O/H⋯Cl contacts, O and N being stronger hydrogen-bond acceptors than Cl. In this family of compounds, N⋯N contacts are, on average, the least favoured, followed by H⋯H, N⋯Cl and Cl⋯Cl. For the molecules with the highest H content (*S*_H_ = 45–50%), *E*_HN_ and *E*_HCl_ have, on average, similar values of around 1.5.

Concerning N⋯Cl halogen-bonding, *E*_NCl_ is spread very widely between 0 and 1.4 and tends to decrease with increasing *S*_H_ due to competition with H⋯N and H⋯Cl contacts (Fig. S10). *E*_NCl_ tends to increase with increasing *S*_N_ (Fig. 15 ▶ *b*) and *S*_Cl_. N⋯Cl contacts have a higher propensity to occur than O⋯Cl interactions (Fig. 12 ▶).

The molecule PECTUO (C_6_H_4_N_3_Cl; Yuan *et al.*, 2012 ▶), with the highest *E*_NCl_ = 1.4, has a unique Cl atom which is indeed involved in ‘halogen bonding’ with the electron lone-pair of an N atom; the N—Cl⋯N angle of 179° is almost flat and the Cl⋯N distance of 2.82 Å is shorter than the sum of the van der Waals radii. In molecule QEDCAF (C_6_HNCl_6_; Zhu *et al.*, 2012 ▶), with *E*_NCl_ = 1.14 larger than unity, the unique N atom interacts with several Cl atoms, but these contacts are of a van der Waals nature. The two closest Cl⋯N distances are 3.59 Å, while the N—lone-pair⋯Cl angle of 136° is far from flat.

### 3.14. CHBrCl aliphatic compounds   

**Figure 16:**
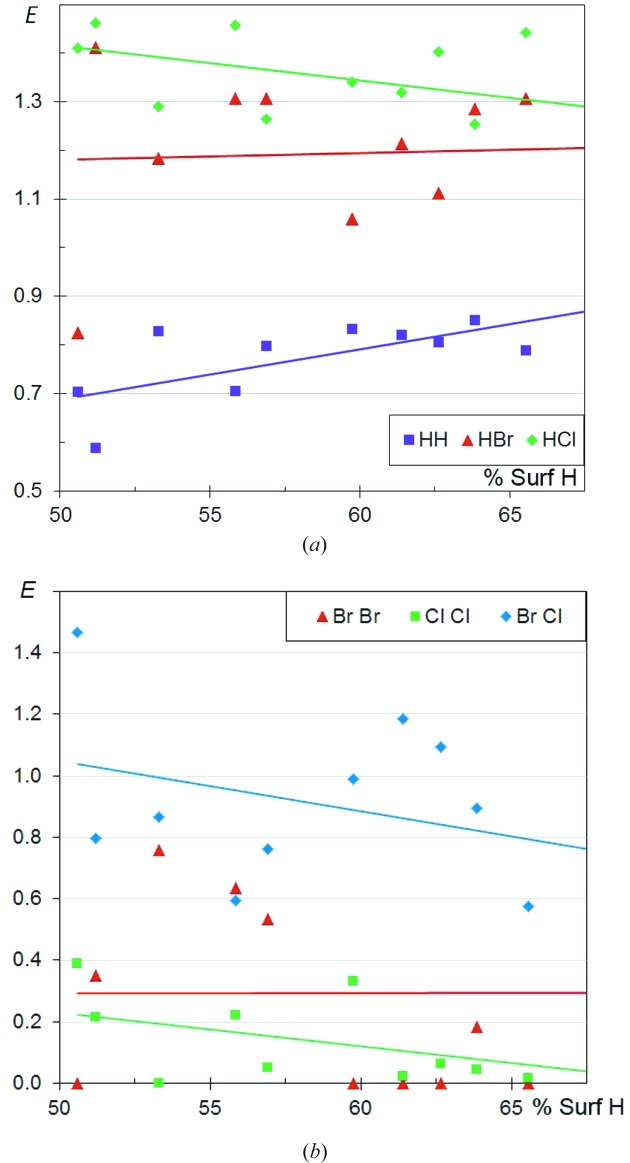
Contact enrichment ratios in crystals of CHBrCl aliphatic compounds as a function of H proportion on the Hirshfeld surface. (*a*) Contacts involving H; (*b*) other contacts.

In order to compare the contact-forming propensity of two different halogen species present in the same molecule, the crystal structures of a series of CHBrCl aliphatic compounds are analysed in Figs. 16 ▶(*a*) and 16 ▶(*b*). The different types of halogen⋯halogen interaction show very clear features. The mixed Br⋯Cl contacts (Fig. 16 ▶ *b*) can be enriched and have the highest likelihood of occurring, with *E*_BrCl_ between 0.6 and 1.43. On the other hand, Cl⋯Cl contacts followed by Br⋯Br contacts are the most disfavoured, with 〈*E*_ClCl_〉 = 0.1 and 〈*E*_BrCl_〉 = 0.3, and many zero enrichment values. When the ‘weak H⋯halogen bonds’ are compared, the H⋯Cl contacts (Fig. 16 ▶ *a*) have a higher propensity to occur than H⋯Br, which is in accordance with the greater electronegativity of Cl than Br. The competitivity between H⋯Cl and H⋯Br contacts can be analysed within the same sample of CHBrCl compounds (Fig. 16 ▶ *a*), and also by comparing CHBr and CHCl molecules (Figs. 1 ▶ and 5 ▶).

### 3.15. CHFCl aliphatic compounds   

**Figure 17:**
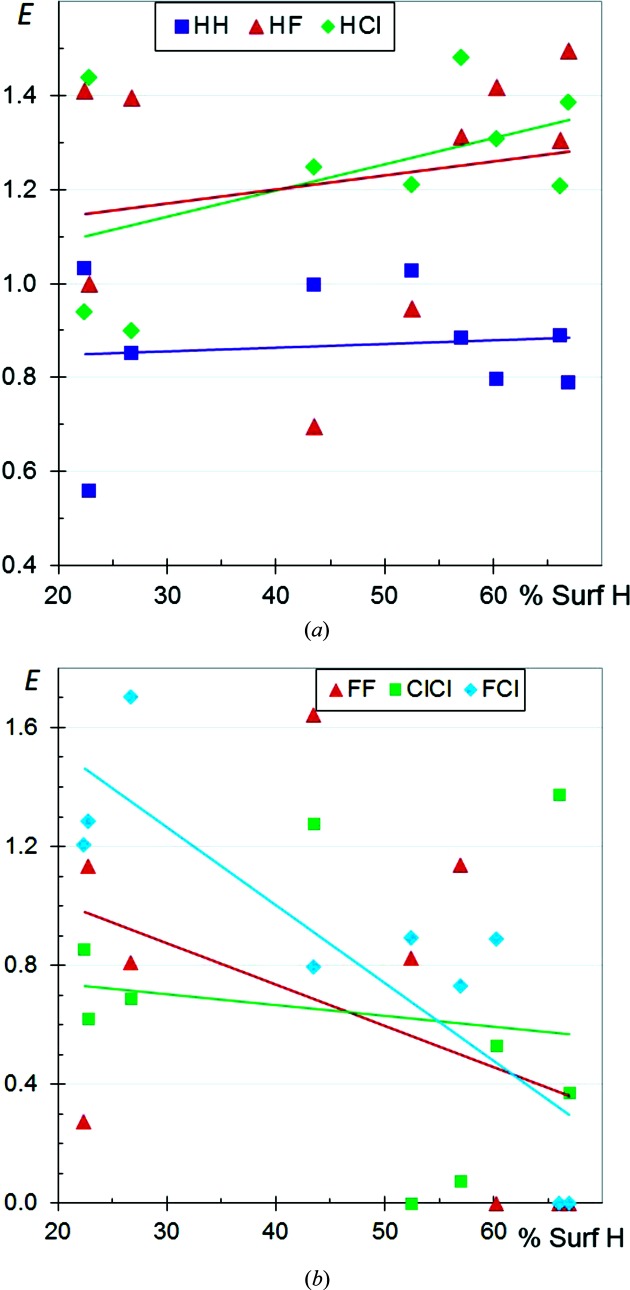
Contact enrichment ratios in crystals of CHFCl aliphatic compounds. (*a*) Contacts involving H; (*b*) contacts within halogen atoms F and Cl.

The contact propensities of F and Cl are analysed in the context of CHFCl aliphatic compounds in Fig. 17 ▶. The three types of halogen⋯halogen contacts show a wide range of enrichment ratios between 0 and 1.7. The mixed F⋯Cl contacts appear very slightly favoured over F⋯F and Cl⋯Cl contacts. However, this preference for mixed contacts is much less pronounced than in the case of CHBrCl molecules. The occurrence in the CSD and the stereochemistry of different types of *X*⋯*X* and *X*⋯*Y* interactions between halogen species was reviewed by Pedireddi *et al.* (1994 ▶). Concerning halogen⋯H interactions, the F and Cl elements show similar trends with enrichment ratios of around 1.2.

## 4. Conclusions   

The molecular Hirshfeld surface in a crystal is representative of the region in space where molecules come into contact. Therefore, its analysis gives the possibility of obtaining quantitative insights into the nature of intermolecular interactions in the crystalline state. The properties of contacts in several series of halogenated organic compounds were statistically analysed using *CrystalExplorer*. Scatterplots of contact enrichment ratios *versus* surface content in a chemical species yield information on the favoured contacts being formed and their dependency on the chemical composition of the mol­ecule. Synthons recurrent in crystal structures can be identified in this way.

All of the H⋯halogen contact types are favoured, displaying on average high enrichment values stable around 1.3–1.7. This indicates that the H⋯*X* contact is a favourable interaction which contributes to the stability of crystal structures, especially in the absence of other hydrogen-bond acceptors (Chopra & Guru Row, 2011 ▶). The two most electro­negative elements, F and Cl, were found to have the highest *E*_H*X*_ ratios. When O is present in a molecule, C—H⋯*X* contacts are still favoured with the concomitant formation of O—H⋯O hydrogen bonds. Analysis of intermolecular contacts in aromatic halogenated compounds confirmed previous findings that C⋯C contacts can be very enriched due to electrostatically favourable parallel π–π stacking between heterocyclic cycles (Jelsch *et al.*, 2014 ▶). C atoms bound to a halogen or not (C_F_ and C_H_) were distinguished in the case of CHF compounds; the two types of C atom show no clear difference in crystal contact formation.

The nature of *X*⋯*X* halogen contacts has been an important matter of interest in crystal engineering. However, generally halogen⋯halogen interactions appear disfavoured in crystal structures. These *X*⋯*X* contacts are more likely to form between the two most polarizable and least electronegative species, Br and I. It was found that unsymmetrical interactions such as Cl⋯Br and, to a lesser extent, F⋯Cl are more likely to form than the corresponding symmetrical *X*⋯*X* contacts. Halogen bonding (*X*⋯O, *X*⋯N and *X*⋯C) where a halogen atom interacts with a hydrogen-bond acceptor appears generally disfavoured, due to competition with H⋯*X* interactions. However when H is scarce in mol­ecules rich in halogen, O or N, the likelihood of halogen bonding increases and can even be favoured in some cases, for example in CHNCl molecules.

The statistical analysis tool presented here uses a limited amount of information on the crystal packing. Nevertheless, additional properties of the molecules, such as the dipole moment, shape or size, could be included. The analysis of outliers or of molecules at the extremity of the graph (*e.g.* small *S*_H_, large *S*_Br_ *etc.*) enables diverse situations to be highlighted, for instance the importance of dipole-moment alignment. Other factors, such as the multipolar *versus* dipolar character of the molecular electric moment or the ratio between unit-cell parameters (*a*, *b*, *c*), also have an influence on electrostatic interactions occurring in a crystal packing. A multivariate principal component analysis (PCA) using more descriptors could yield sharper trends with higher correlation and some outliers may be better fitted. Such an extended methodology has the potential to unravel novel relationships concerning packing contacts, molecular properties and crystal parameters.

## Supplementary Material

Lists of CSD codes and scatterplots. DOI: 10.1107/S2052252515003255/bi5042sup1.pdf
