# Supplementary material for: Likelihood of atom–atom contacts in crystal structures of halogenated organic compounds
Source: IUCrJ. 2015 Apr 10;2(Pt 3):327–40. doi: 10.1107/S2052252515003255 (PMC4420543; doi:10.1107/S2052252515003255)

IUCrJ

Volume 2 (2015)

Supporting information for article:

Likelihood of atom-atom contacts in crystal structures of halogenated organic compounds

Christian Jelsch, Sarra Soudani and Cherif Ben Nasr

Supplementary Materials

Likelihood of crystal contacts between chemical species
for halogenated organic compounds.

Jelsch C., Soudani S. & Ben Nasr C.

List of compounds retrieved from the Cambridge Structural Database included in each family of molecules.

LIST of CHCl aliphatic compounds

SEGWIK HCCYHE01 PCLCHX10 PEDGAI GOGXEF OCLOCT XOQLAR XILWEU PCCHLD TOCTCD ZAJLEB TEVQAN RIFWIM RIFWOS TUWBOC HEVNAX CADINC01 REZHIO FITVIN LESXOY YOCVUI RIQTOA GIQHIX RIQTUG

LIST of CHCl aromatic compounds

PNCLBZ TCLBEN02 TCHLBZ ABUMIT DCLBEN01 MCBENZ YAFHUI TCNAPH DCLNAQ DUXRAP HCLBPH IRUYIE KASJIY POGNUU DAWRUO FUHDAN01 GUQYOG DCLBIP SIFGIX DCLANT10 DMCPAY FIXWUE ZZZBLP01 CTCLST QQQFED02 LUXPUQ LIYPOZ FAHTEO FECZOE OVIRAN NEHZAD ELINUI

LIST of CHF aliphatic compounds

AMECIE OCFCHX10 ADAREE BOMJIW DEZDUH DABLIB TELSEI TELSAE QEFDIO

LIST of CHF aromatic compounds

CUWYUP FACJAU ZELDOJ PUGDEB PAVVAWA01 FACFOE FAGGEV FACFAQ AXUBUR01 AXUCEC01 CAXNUL DFNAPH10 FLNAPH BAWPUK FELLOY DEXWOU SERTOA DARXOK ATEVEB SETTOC AXIDER APUDEV

LIST of CHBr aliphatic compounds

BROFRM05 BAVJUD GOPNEE FOXJUY IFOKOD IJUHAX FEHCOK EVEYAG BAHZEP HESCUD BATYUR BATWUP DATPAQ FUTXUO HADKEC BCYLON10 BHPCHD10 COGTUN DAQTUK FEXDIW FITVEJ DEZLEZ IZAMUR

LIST of CHBr aromatic compounds

TETBBZ02 DEXXAG QOLLOS BRNPHL COXLOQ DAWSEZ FUBPUO HIQQON REBMES DEFBIA HOYWIB SECHOZ SECTEB QQQFDS02 GEQPOJ CARJEM DOTRUA MEGTOI DAHNEF FAHTIS IVUJIT PORRAQ KUSMAN JOGMUO FABSIK10 GAVBUB OKANOE APOWOS

LIST of CHI aliphatic compounds

HEHVEX DICHEP VADBUY GIHUTIA QIBMOE KANGUB DITCDC WAHRAY WAHREC XIFCIA IPCUND WAHLUM BOBLAG XUPMIE GISWAH LEDYIE TANRAB XEPPAJ IMTCOS

LIST of CHI aromatic compounds

REBBEG REKYAI TIGGUI ZZZPROO01 SOYGET POWNEV SAQZOY01 BOBGUV JEVMON NIFHAM XAMBAP ZZZSLE01 IVUHEN EFALUU KUZFAN IVUHAJ KEGXEA LITBIA MEBCOL WUMVOQ

LIST of C H Cl O aliphatic compounds

CLACET02 LIQVOW DISZIO PAMPEY CLSUCC CDCDOX PRCHLA CLCYHX GANKIP CEFZIX CUGNUO CUGPAW CUGPEA CUGPIE CUGPUQ CUGQAX CUGQEB CUGQIF CUGQOL HOYCOO HOYCUU POPFUV CLDTOL PRCHLA

LIST of C H F O aliphatic compounds

MEWZOF VEVSIZ IPUNEN MIJPUQ SINYUK SITFOR POMXEV BUYKOW ESIFIV PIBXUT OYAGEB BAPFEF BAPFIJ BAPFOP BAPFUV BAPGAC BAPGEG BAPGIK BAPGOQ

LIST of C H Br O aliphatic compounds

BRMACA CURLEG EQUTOA SEDGOZ SAZSES WOCHIF WANNEE PUZNAA QERVIS QERVOY WUQDOC WUQDUI YIVNEX JINYOV VAHZEJ BRDTOL

LIST of C H N Cl aliphatic compounds

VITRUL FELPIV ETEYAE CLTRZL BOTYIT DCPYAZ AMCPYZ GAPPIY NIKSIK PUGWEU NUNKUC QEDCAF UKIWOB PIJBOO HCZCHP PECTUO QECRUN XIXREC DCHLAN01 IJUTUD

LIST of C H Br Cl aliphatic compounds

FEMLAK UROWEE VIOLAC DEKHAC VEQKOR SAFFIO YICLIF AFACIU MUKHUV WUGHAH HIJVIF IDOKOD

LIST of C H F Cl aliphatic compounds

FAVTAX XEZHAM XEZHEQ PICVUS KEQNAW JALLAK CLFBZD FPCLET GEFPAI

Figure Sup1. Enrichment ratios in CHF aromatic compounds as a function of fluorine proportion S_F on the molecular surface.

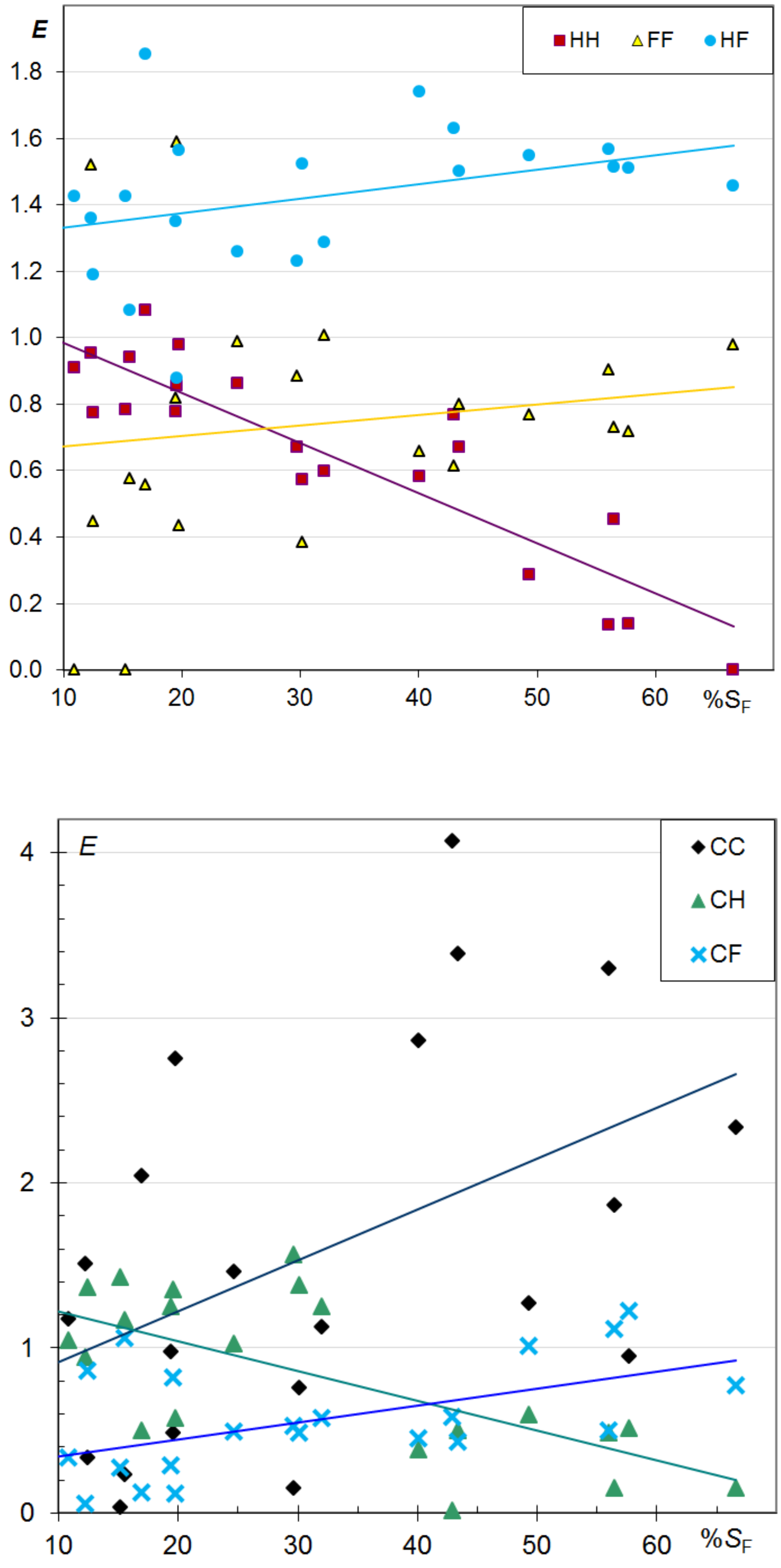

Figure SUP2. Enrichment ratios for the different C···C contacts in CHF aromatic compounds. Carbon atoms bound to F (Cf) or not (Ch) are differentiated.

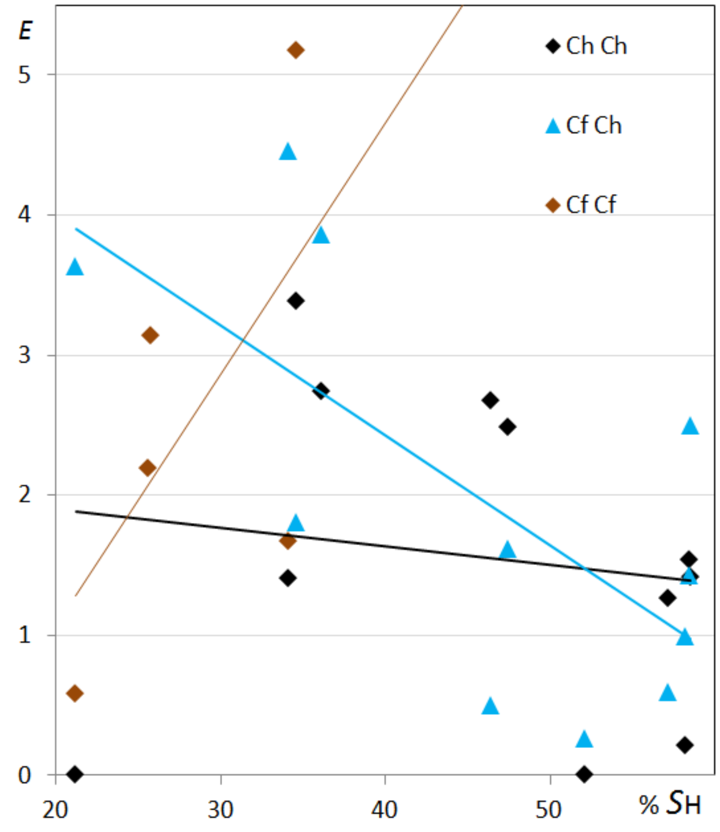

Figure Sup3. Alternative scatterplot of enrichment ratios in CHBr aromatic compounds as a function of bromine proportion S_{Br} on the molecular surface.

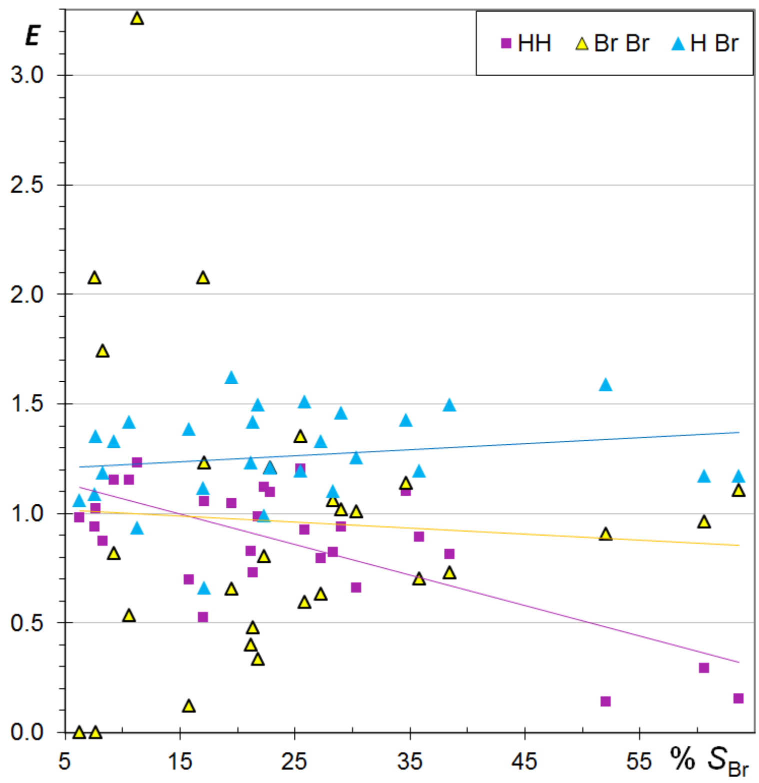

Figure Sup4. Alternative scatterplots of enrichment ratios in CH I aromatic compounds as a function of carbon and iodine proportion on the molecular surface.

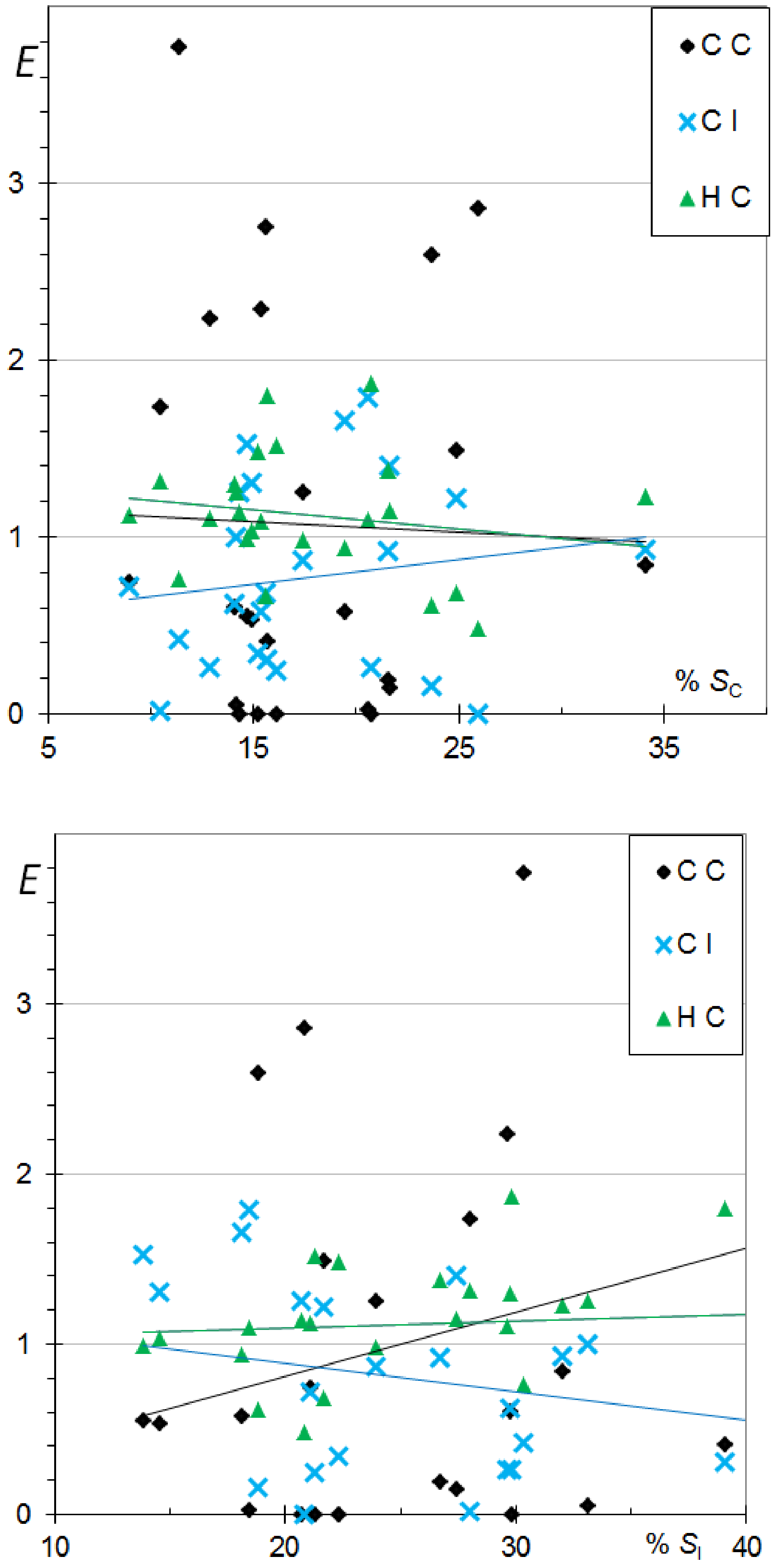

Figure Sup5. Comparison of hydrogen···halogen contact enrichment ratios in the different halogenated CHX compounds (aliphatic and aromatic).

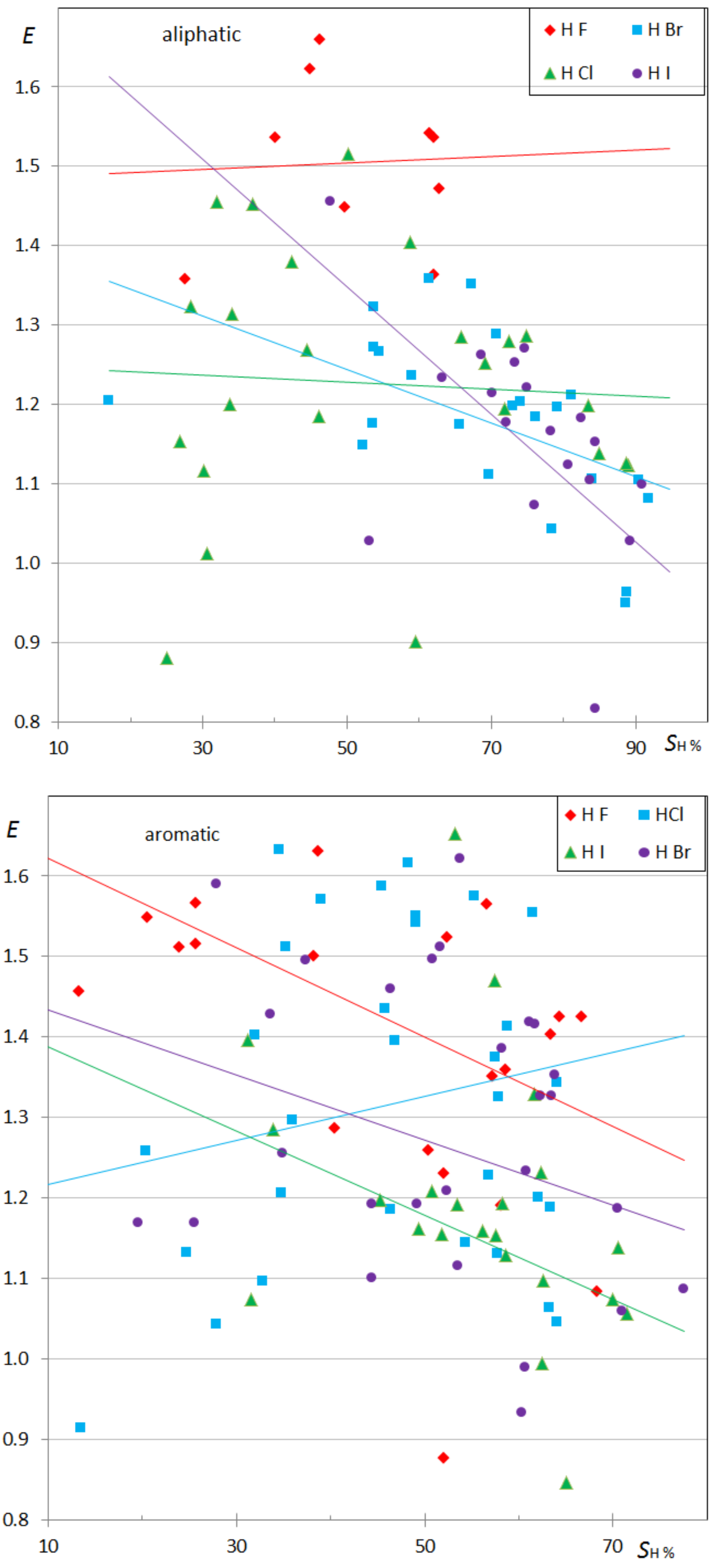

Figure Sup6. Scatterplots of enrichment ratios in C H O Cl aliphatic compounds as a function of oxygen and chlorine proportion on the molecular surface.

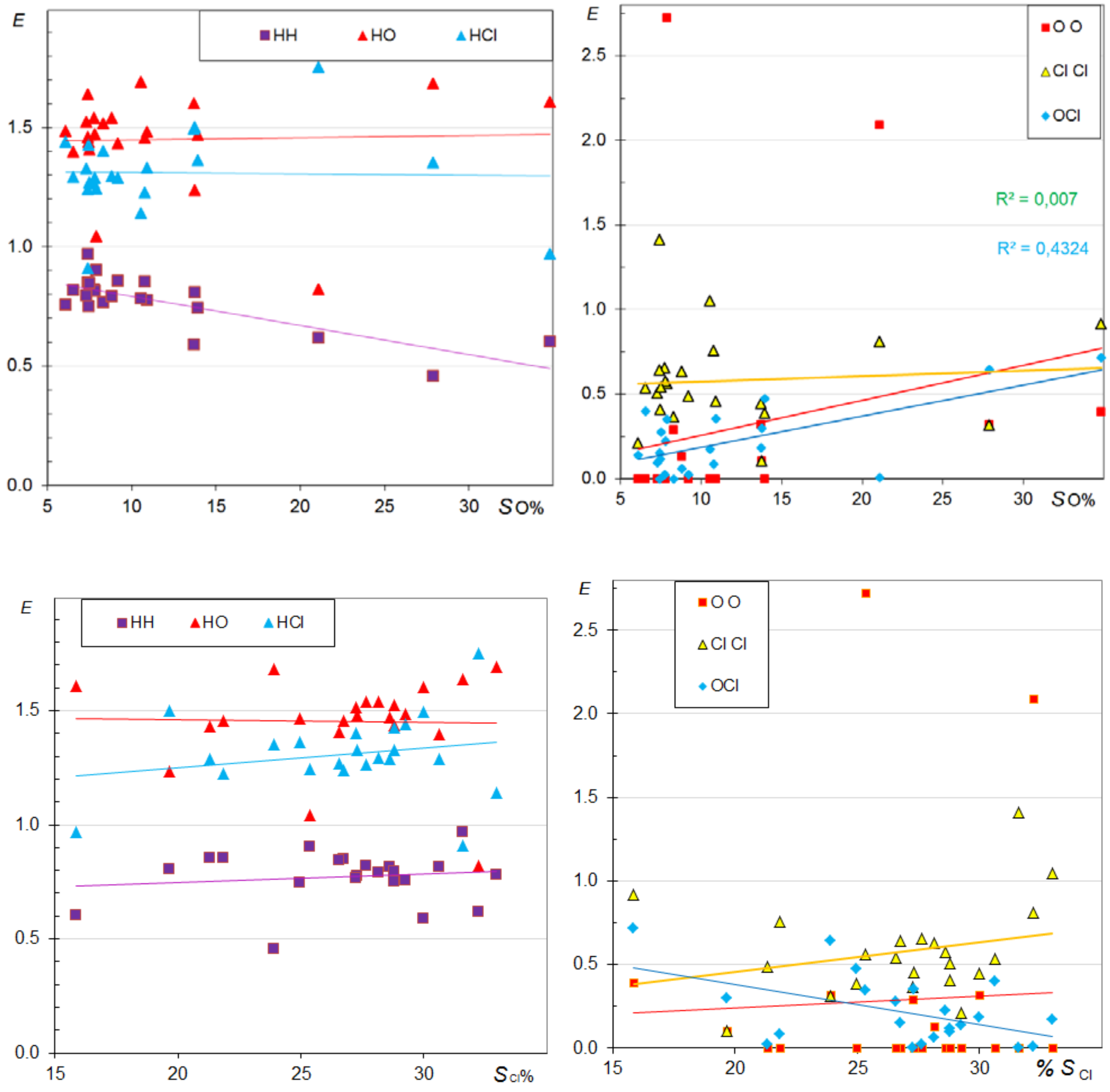

Figure Sup7. Scatterplots of enrichment ratios in C H O F aliphatic compounds as a function of oxygen and fluorine proportion on the molecular surface.

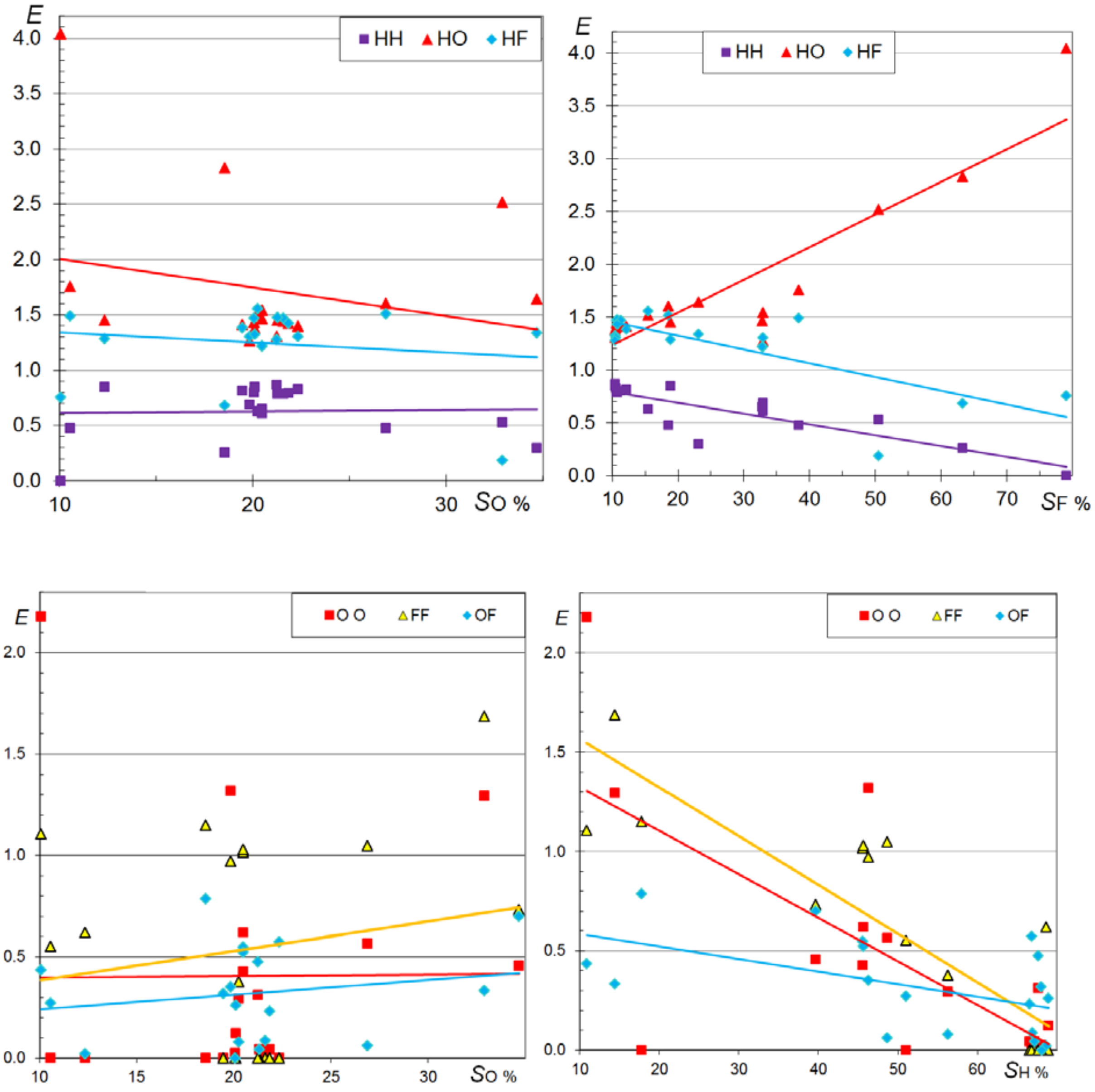

Figure Sup8. Preferential formation of O-H···O and C-H···F interactions in the crystal of compound 2,2,3,3-Tetrafluorobutane-1,4-diol (reference code POMXEV).

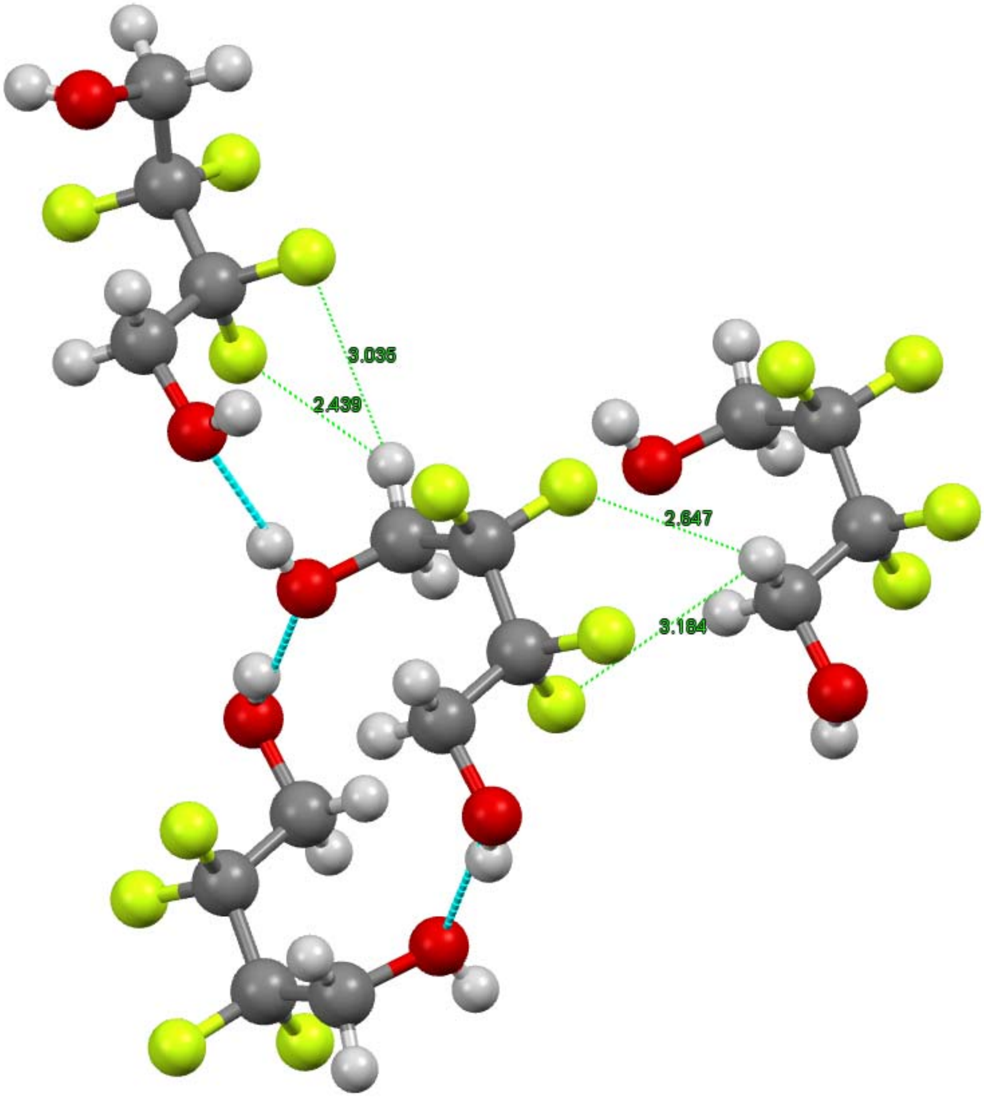

Figure Sup9. Alternative scatterplots of enrichment ratios in CHOBr aliphatic compounds as a function of different chemical proportions on the molecular surface.

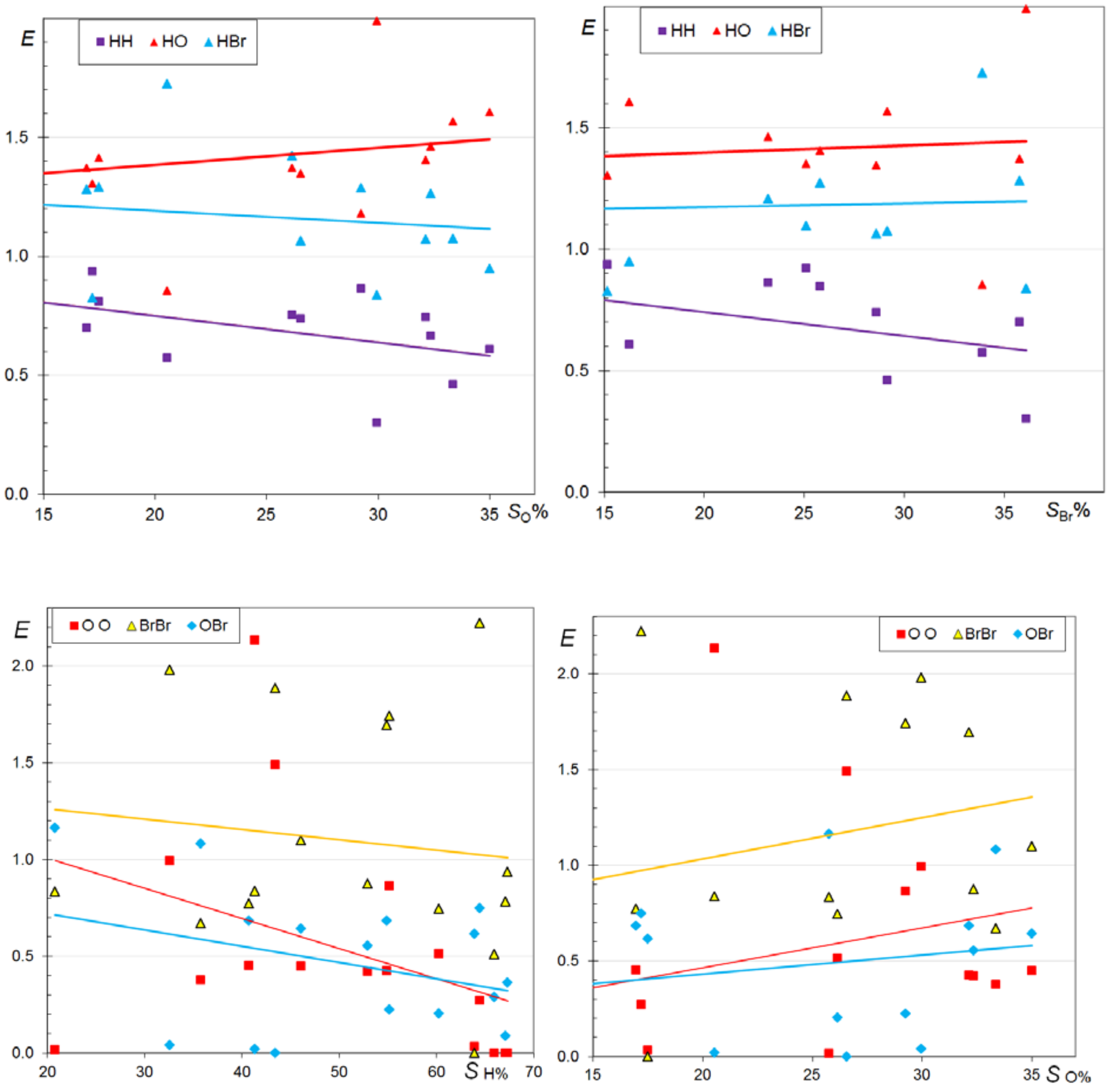

Figure Sup10. Alternative scatterplots of enrichment ratios in CHNCl aliphatic compounds as a function of different chemical proportions on the molecular surface.

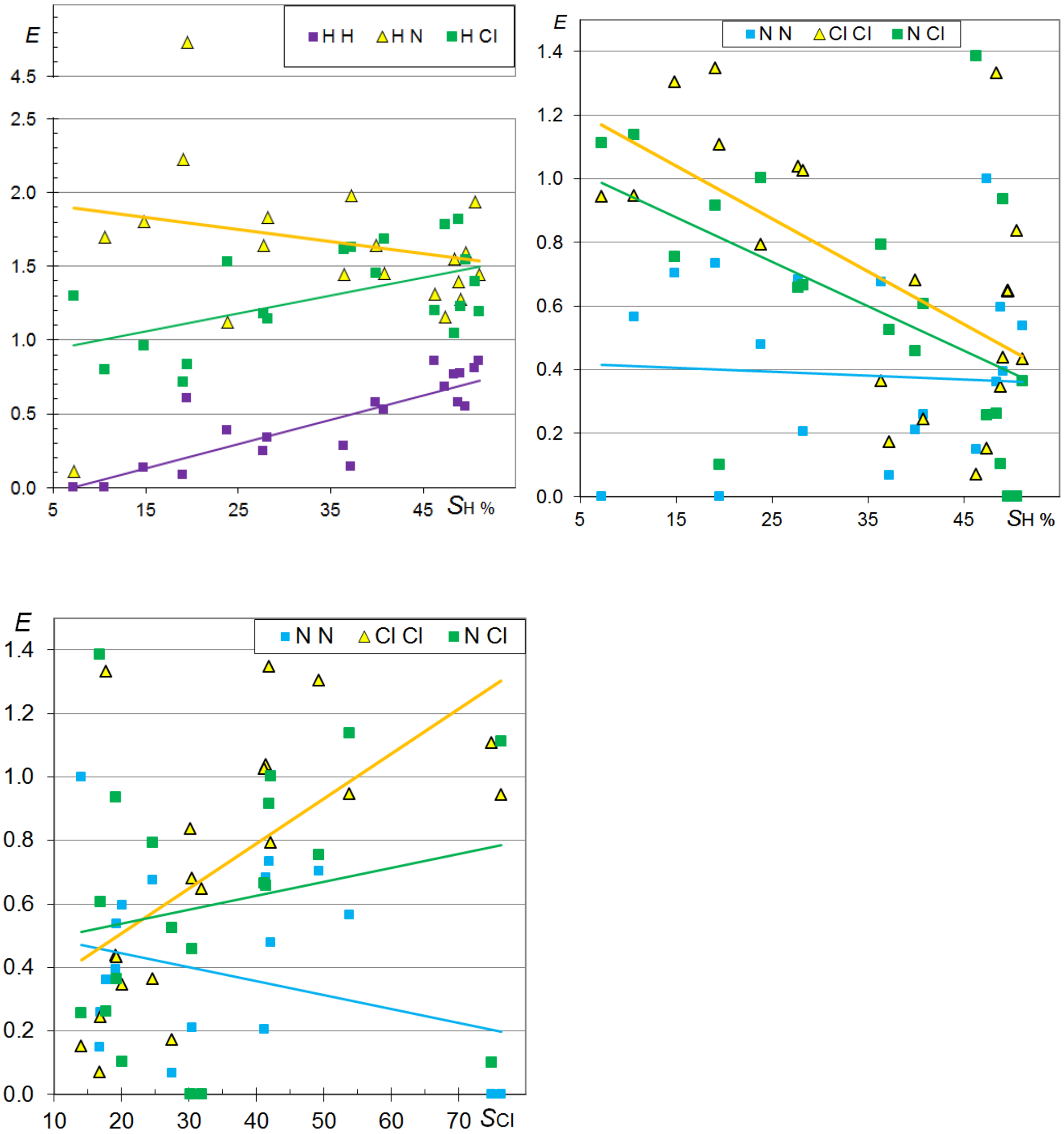

Supplement: Supplementary file 1 [file m-02-00327-sup1.pdf]
